# Prospective evaluation of artificial intelligence integration into breast cancer screening in multiple workflow settings: the GEMINI study

**DOI:** 10.1038/s43018-026-01126-1

**Published:** 2026-03-10

**Authors:** Clarisse Florence de Vries, Gerald Lip, Roger Todd Staff, Jaroslaw Artur Dymiter, Benjamin Tse, Annie Ng, Georgia Fox, Cary Oberije, Lesley Ann Anderson

**Affiliations:** 1 Glasgow Lab for Data Science & AI, Public Health, School of Health and Wellbeing, University of Glasgow, Glasgow, UK; 2 National Health Service Grampian, Aberdeen, UK; 3 Grampian Data Safe Haven (DaSH), University of Aberdeen, Aberdeen, UK; 4 DeepHealth Inc., Somerville, MA USA; 5 Kheiron Medical Technologies Ltd., London, UK; 6 Biostatistics and Health Data Science, Institute of Applied Health Sciences, School of Medicine, Medical Sciences & Nutrition, University of Aberdeen, Aberdeen, UK; 7 Interdisciplinary Institute, University of Aberdeen, Aberdeen, UK

**Keywords:** Breast cancer, Cancer screening, Cancer, Computational biology and bioinformatics

## Abstract

Artificial intelligence (AI) tools can improve breast screening performance but different screening sites have varying needs. Here the GEMINI prospective evaluation of 10,889 women, within one UK region, used both live AI integration and simulations to model 17 different ways AI could be used in breast screening. All women received routine care. One AI tool was assessed. When the AI tool recommended recall but routine double reading did not, cases underwent additional human review, detecting 11 additional cancers. The primary AI workflow could improve cancer detection by 10.4% (1 per 1,000), maintain the recall rate (0.8% reduction) and reduce workload by up to 31%. Other workflow variations significantly improved all measured metrics (superiority in cancer detection rate, recall rate, positive predictive value (PPV), sensitivity and specificity) with up to 36% workload savings. Different AI integrations in breast screening could offer various clinical and operational gains, allowing for adaptation to local healthcare needs.

## Abstract (web summary)

De Vries et al. performed prospective evaluation of AI for breast cancer screening leveraged in many different workflow settings and report several clinical and operational gains depending on the type of AI integration.

## Main

Artificial intelligence (AI) tools improve breast screening by reducing workload and maintaining or improving cancer detection rates (CDRs)^1–4^. AI is now being used in some clinics in the United States^5^, Hungary^4^ and within the Danish Capital Region breast cancer screening program^3^.

Most studies evaluating AI performance have used historical data^6,7^. These retrospective studies have limited ability to assess how AI may influence clinical practice, and specifically, improve cancer detection. Recent prospective studies have demonstrated that AI use in breast screening can be safe and effective, increasing cancer detection and reducing workload^1,2,8^. The Swedish ScreenTrustCAD study reported that double reading by one radiologist plus AI resulted in 4% more screen-detected cancers and a 4% lower recall rate than routine double human reading^1,2,8^. The Swedish MASAI randomized controlled trial reported in its clinical safety analysis (*n* = 80,033) that AI-supported screening (single or double reading based on AI risk score) detected 1 per 1,000 more cancers and reduced workload by 44.3% compared to routine screening^8^. Similarly in Denmark, AI-assisted reading increased CDR by 1 per 1,000, while reducing the recall rate by 20.5% and reading workload by 33.5%^3^.

Despite these promising results, the demonstrated improvements depend on the AI tool and how it is integrated into the screening pathway. Randomized controlled trial methods limit assessment to one or two implementation strategies, while AI can be used in various configurations to accommodate different performance requirements. In addition, breast screening sites have varying clinical and operational needs. Thus, evidence to date provides a limited perspective of the benefits of AI use in breast screening.

The prospective service evaluation presented, Grampian’s Evaluation of Mia in an Innovative National breast screening Initiative (GEMINI), combines both live AI integration and workflow simulations to enable the assessment of multiple AI implementation strategies. The design was guided by the preferences of women attending screening^9^ and human screen readers^10^, with AI supporting, not replacing, human decision-making.

A commercially available AI system (Mammography Intelligent Assessment (Mia) v.3, Kheiron Medical Technologies Ltd) was prospectively used within a routine breast screening service, using two complementary approaches: (1) as an ‘AI-Additional Read’ to increase cancer detection, flagging suspicious cases not recalled by routine double reading for additional human review; and (2) for triage to reduce workload. For this approach, AI ran prospectively, in the background, to enable simulations of AI as a second reader.

A secure evaluation workspace ensured that the analyses were performed independently from the AI vendor. Triage workflows were modeled separately and in combination with the AI-Additional Read workflow to evaluate the impact of AI on cancer detection, recall rates and workload reduction. Seventeen different ways in which AI could be used in breast screening were assessed. These included two AI-Additional Read workflows (one live, one simulated), five triage and ten combinations of both approaches. All workflows were specified a priori, with the primary workflow aiming to optimize CDR while reducing workload.

This innovative prospective design enabled modeling and evaluation of multiple pathways for AI integration in breast screening. This provides a comprehensive view of how AI can meet various breast screening programs’ clinical and operational needs.

## Results

### Cohort characteristics

**Fig. 1:**
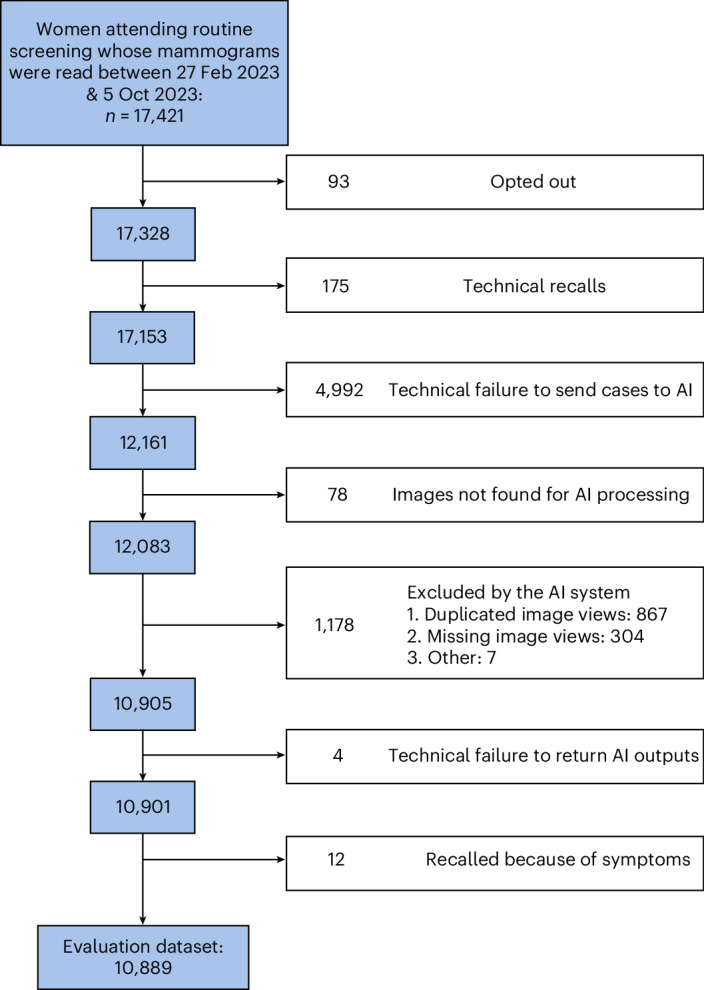
The diagram shows the flow of screening attendees through to study inclusion. Blue boxes indicate retained participants; white boxes indicate exclusions.

The UK National Health Service (NHS) Breast Screening Programme invites women aged 50–71 years, every 3 years, for routine breast screening using digital mammography. Between 27 February and 5 October 2023, 17,421 women attended routine screening at NHS Grampian (Aberdeen, UK) (Fig. 1). Of these, 93 (0.5%) women opted out of the study and 175 (1.0%) technical recalls were excluded.

Images from 4,992 (29.1%) women were not sent for AI assessment because of an unanticipated legacy IT coding error, initiating a mammographer session timeout after 30 min of inactivity (Fig. 1). To evaluate potential bias, the human CDR and the recall rate were compared between cases affected and unaffected by the IT issue. Both did not significantly differ (chi-squared tests: CDR 10.2 versus 10.3 per 1,000, *P* = 1.00; recall rate 4.2% versus 4.4%, *P* = 0.93). The median age for women in both groups was 60 years.

The AI system did not read 1,260 (10.4%) mammogram examinations because of its exclusion criteria. Of the 10,889 women included in the evaluation dataset, 106 (9.7 per 1,000) were diagnosed with cancer within routine screening; 73.3% were invasive (Table 1). An additional 11 cancers were diagnosed with the support of AI.

**Table 1:** Breast screening cohort characteristics

	Number of women (n = 10,889)	Percentage
**Age (years)**		(% of cohort)
50–54	2,512	23.1
55–59	2,858	26.2
60–64	2,525	23.2
65–69	2,187	20.1
70+	807	7.4
**Special requirements**	**704 (6.5%)**	(% of cohort)
Physical requirement	259	2.4
Language needs	140	1.3
Other/special needs	79	0.7
Implants	64	0.6
Wheelchair	41	0.4
Deaf	20	0.2
Learning difficulties	15	0.1
Blind	8	0.1
Two or more special requirements	78	0.7
**Routine screen-detected breast cancers**	**106 (1.0%)**	**(% of routine screen-detected cancers)**
**Histology type**		
Ductal carcinoma in situ (preinvasive)	23	21.7
Invasive breast cancer	83	78.3
**Tumor biology**^**a,b**^		
ER^+^	72	67.9
PR^+^	63	59.4
HER2^+^	12	11.3
Triple negative	4	3.8
**Tumor size**		
<15 mm	49	46.2
≥15 mm	57	53.8
**AI screen-detected breast cancers**	**11 (0.1%)**	**(% of AI screen-detected cancers)**
**Histology type**		
Ductal carcinoma in situ (preinvasive)	4	36.4
Invasive breast cancer	7	63.6
Tumor biology*		
ER-positive	7	63.6
PR-positive	7	63.6
HER2-positive	3	27.3
**Tumor size**		
<15 mm	7	63.6
≥15 mm	2	18.2
Unknown	2	18.2

^a^Tumor biology was only quantified for invasive breast cancers. ^b^For invasive routine screen-detected cancers, ER, PR and HER2 status was not available for 4, 5 and 6 cancers, respectively. ER, estrogen receptor; HER2, human epidermal growth factor receptor 2; PR, progesterone receptor.

### Workflows

**Fig. 2:**
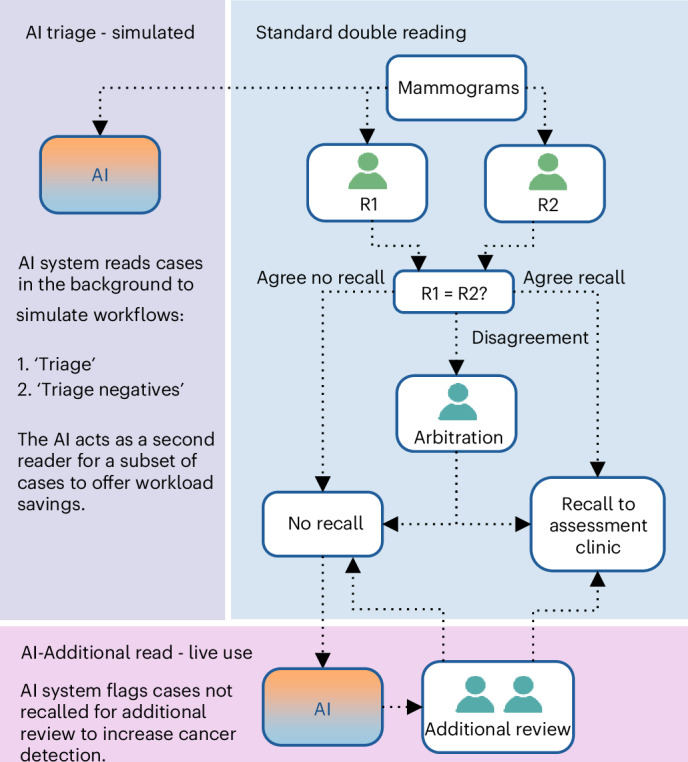
GEMINI study design. Service evaluation workflow of AI integration into routine breast screening pathway. All women underwent standard double reading; AI ran simultaneously. Cases flagged by the AI system but not recalled by the human readers underwent additional review. AI outputs were used to simulate different AI workflows. R1, reader 1; R2, reader 2.

In total, the performance of 17 a-priori-specified AI workflows was assessed (Fig. 2). AI was assessed in different configurations, with varying operating points (OPs). In this study, AI was used with four different OPs (1–4). OPs are vendor-prespecified decision thresholds and offer a trade-off between sensitivity and specificity. OP1 has the highest specificity, while OP4 has the highest sensitivity. The AI system was used ‘live’ as an additional reader at OP2 to offer increased cancer detection. This enabled simulation at a higher specificity, OP1, which would reduce workload relative to OP2.

The AI results (recall opinion and regions of interest) were not available to the human readers during the standard reading and arbitration process. These results were only released to the readers when the AI suggested to recall but routine screening did not (*n* = 1,345, OP2). These mammography examinations were additionally arbitrated, with 55 women (4.1%) recalled for additional investigations.

Most additionally read examinations (*n* = 848; 63.0%) took between 0 and 30 s (Table 2). There was a significant trend with an increasing proportion of women recalled for further investigation with increasing arbitration time (chi-squared test for trend, *P* < 0.001). Of those not recalled (*n* = 1,290; 95.9%) reasons included: a review of prior mammogram images indicating no notable change in appearance since the last mammogram (53.6%); AI region of interest showing benign features (30.2%) or vascular calcification (7.0%). Other reasons for not recalling included surgical scar or area of change because of previous breast surgery (2.6%), no lesions seen (2.4%) and prior assessment of the area of concern found no cancer (1.6%).

**Table 2:** Time duration of the additional review of mammography examinations where the AI suggested to recall but routine breast screening did not

Time duration of additional review (s)	Number (%) of cases additionally reviewed	Number of recalls (% of number reviewed)	Number of cancers (% of cases recalled)
0–30	848 (63.0)	7 (0.8)	3 (42.9)
30–60	357 (26.5)	19 (5.3)	4 (21.1)
60–120	114 (8.5)	19 (16.7)	2 (10.5)
120–300	16 (1.2)	5 (31.3)	2 (40.0)
Time not recorded	10 (0.7)	5 (50.0)	0 (0)

Screening performance when using the AI for triage was simulated in two modes: (1) ‘triage’, where the AI was used as the second reader when the AI and reader 1 had the same recall or no recall opinion, optimizing workload savings; and (2) ‘triage negatives’, where the AI was used as the second reader when the AI and reader 1 agreed not to recall, reducing workload while avoiding additional recalls.

Five solo triage workflows were modeled: the ‘triage’ workflow using OP1 and OP2, and the ‘triage negatives’ workflow using OP2, OP3 and OP4. The two AI-Additional Read and the five triage workflow configurations enabled ten combination workflows to be assessed, including the primary workflow.

**Fig. 3:**
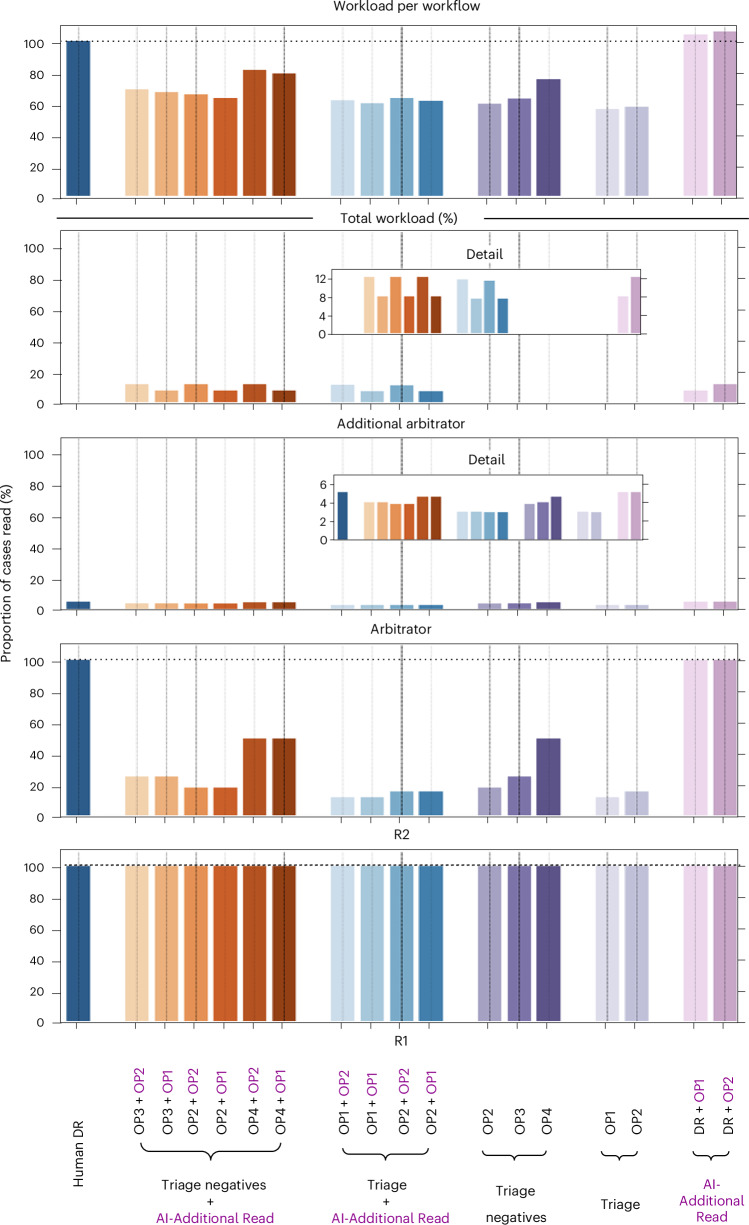
Reading workload varied across the 17 AI workflows assessed. Bar plots of the reading workload for each of the 17 workflows compared to routine double reading (DR) (proportion of cases read), including a breakdown according to reader role. For quantification of the additional arbitration workload, one human arbiter was assumed. R1, reader 1; R2, reader 2. Source data

Table 3 presents the performance metrics across the 17 workflows, with Fig. 3 offering more detail on workload.

**Table 3:**
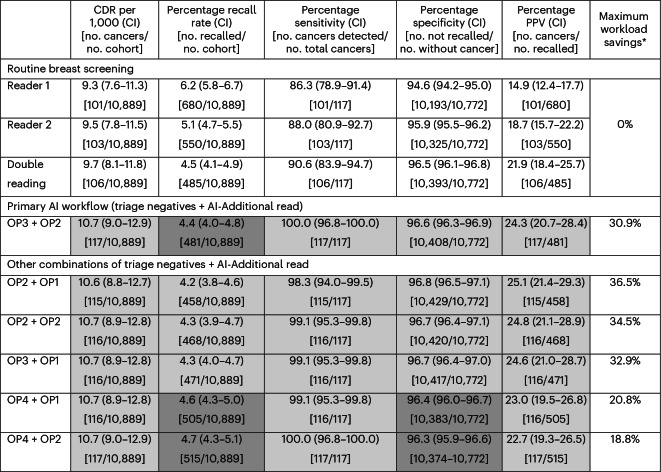
Performance of the primary AI workflow and five other combinations of triage negatives plus AI-Additional Read compared to routine breast screening

For the combination workflows, the first OP relates to the triage/triage negatives workflow; the second OP relates to the AI-Additional Read workflow. ^a^Maximum workload savings are derived by combining the number of reads reduced for reader 2 and arbitration, and increased for additional arbitration (based on one human arbiter). A-priori-defined tests were performed for all combination AI workflows compared to routine double reading for CDR, recall rate, sensitivity, specificity and PPV. Test results are indicated with cell shading: superior (light gray); non-inferior (medium gray), inferior (dark gray; none in this table). No cell color indicates that no test was performed. AI, artificial intelligence; CDR, cancer detection rate; CI, confidence interval; PPV, positive predictive value.

#### The primary AI workflow: combining increased cancer detection with workload savings

The primary AI workflow, combining the simulated AI ‘triage negatives’ workflow (OP3) with the live AI-Additional Read workflow (OP2), increased the CDR (1.0 per 1,000, 10.4% relative increase) while maintaining the recall rate (0.8% relative decrease) (Table 3).

Of the 11 additionally detected breast cancers, seven were invasive (6 grade 2, 1 grade 3). The remaining four were ductal carcinoma in situ (three high-grade, one intermediate-grade). Four of the 11 cancers had already undergone arbitration after routine double reading before the AI-assisted review.

Consensus was determined by two or three human arbiters during the GEMINI service evaluation. The number of human reads would decrease by 31% if consensus was determined by one arbiter, 25% by two and 19% by three arbiters. Overall, the primary AI workflow would achieve enhanced cancer detection and workload savings without increasing recall rates (Table 3).

Superiority was shown for CDR, sensitivity, PPV and specificity. This remained unchanged after a sensitivity analysis, assuming nine instead of 11 additionally detected cancers.

#### Other workflows combining increased cancer detection with workload savings

Clinical sites may seek different trade-offs between CDR, recall rate and workload savings. Three of the nine remaining combined workflow strategies demonstrated superiority across all tested metrics. These combined the ‘triage negatives’ and AI-Additional Read workflows (OP3 + OP1, OP2 + OP2, OP2 + OP1), increasing CDRs by 8.5–9.4%, reducing recall rates by 2.9–5.6% and saving 33–36% of the workload (based on using one arbiter) (Table 3). However, these workflows did not detect all cancers identified through the primary AI workflow (Table 3). Other workflow combinations of ‘triage negatives’ or ‘triage’ with AI-Additional Read would increase CDRs (by 6.6–10.4%) and decrease workload (quantified as the number of human reads) by 19–40.1%, but increase recall rates (by 2.7–12.0%) (Tables 3 and 4).

**Table 4:**
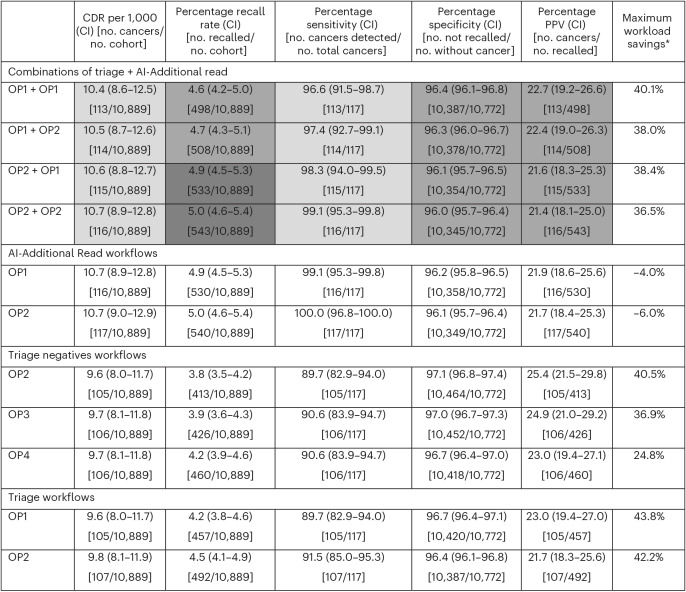
Performance of the remaining 11 workflows (four triage + AI-Additional Read, two AI-Additional Read alone, three triage negatives alone, two triage alone)

For the combination workflows, the first OP relates to the triage/triage negatives workflow; the second OP relates to the AI-Additional Read workflow. ^a^Maximum workload savings are derived by combining the number of reads reduced for reader 2 and arbitration, and increased for additional arbitration (based on one human arbiter). A-priori-defined tests were performed for all combination AI workflows compared to routine double reading for CDR, recall rate, sensitivity, specificity and PPV. Test results are indicated with cell shading: superior (light gray), non-inferior (medium gray), inferior (dark gray). No cell color indicates that no test was performed. CDR, cancer detection rate; CI, confidence interval, PPV, positive predictive value.

#### AI-Additional Read workflows: increased cancer detection

The live AI-Additional Read workflow (OP2) increased cancer detection but, as expected, led to increased workload (6%) and recall rates (11.3%). Simulating the AI-Additional Read at OP1 resulted in lower increases to the recall rate (9.3%) and workload (4%) but would have resulted in one less AI-detected cancer (Table 4).

#### AI triage workflows: workload savings

For healthcare settings wanting to reduce workload, using AI for triage enables single human reading for a subset of cases where AI can serve as the second reader. Compared to routine screening, one modeled ‘triage’ workflow (OP1) would result in the highest workload savings of 44%, with a 5.8% reduction in the recall rate (Table 4). PPV increased by 5.1%, with one cancer missed compared to routine double reading (Table 4). A ‘triage negatives’ workflow (OP3) would reduce workload by 37% and the recall rate by 12.2% while identifying all cancers detected by routine screening. The PPV would increase by 13.8%, resulting in the highest reduction of false positives (Table 4).

## Discussion

The methodological approach used in the GEMINI study demonstrates multiple AI implementation strategies that could improve breast screening programs. The primary AI workflow was superior to routine double reading for cancer detection (1 per 1,000, 10.4% increase) without increasing the number of women recalled for further investigation. Workload savings of up to 31% could be achieved. Other workflows assessed show that AI could be adjusted to fit the different needs of breast screening programs and sites. Sites could select a workflow based on their priorities, such as detecting more cancers, reducing recalls or saving on workload, to find the option that best aligns with their operational goals and capacity.

Similarly to previous prospective studies^1,2,8^, our study showed that AI can increase cancer detection while decreasing workload, demonstrating the generalizability of these findings to a UK screening population. The primary AI workflow demonstrated a similar additional CDR (1 per 1,000) to the Swedish MASAI trial, which used AI to triage screening examinations^8^. In the MASAI trial, workload was reduced by 44%. In Denmark, where AI is routinely used in one region, workload has been reduced by 33.5% and recall rates by 20.5%^3^. In the GEMINI study, the primary workflow could enable up to 31% workload savings without increasing the recall rate. Higher workload savings could be achieved using other implementation strategies, which also reduce recall rates without affecting the number of cancers detected through routine screening. Despite different AI systems, clinical settings, screening pathways and study methods, which limit direct comparisons, all studies show beneficial results of using AI in breast screening. The GEMINI study adds evidence to the literature by enabling multiple AI strategies to be evaluated and providing a more complete picture of the available AI implementation options.

Compared to a previous retrospective study conducted at the same site^11^, the routine CDR was higher (9.7 per 1,000 versus 8.0 per 1,000) during the GEMINI study, while the recall rate was lower (4.5% versus 5.0%). This could be explained by variation in baseline cancer incidence. Another cause may be the delays in breast screening due to the coronavirus disease 2019 pandemic, leading to longer screening intervals (>3 years)^3^. The human experts’ reading practice may also have been influenced by their awareness of and participation in this study, which may have increased the center’s routine double reading performance.

This prospective service evaluation using AI live in the UK was limited to evaluating one AI system within a single UK region. The study controlled for radiologist–AI interaction by only releasing the AI opinion after the final routine double reading decision to the additional arbitration readers, allowing for a true reflection of the contribution of AI in increasing cancer detection. A 3-year follow-up of women in the study for interval cancers is not yet available, meaning that the actual sensitivity of the human readers and AI system could not be assessed. We plan to monitor the interval cancer rates in this cohort using the standard audit pathway in our participating screening center. Changes to the mammography imaging machines (hardware and software) were paused during the study because prior work indicated that such changes may affect AI performance^11^. The triage workflows were simulated, meaning that human behavioral changes in response to AI use in the screening workflow could not be fully assessed. In addition, simulating the workflows implicitly assumes that changes in workload do not affect reader performance. Future studies using the optimal workflows investigated in the GEMINI study would allow further assessment of AI use in clinical practice. The AI excluded a relatively high rate of mammography examinations (10.8%) that fell outside of its intended use compared with previous studies using different AI tools^2,8^. The reported performance improvements are not applicable to women ineligible for AI reading; therefore, they would appear smaller when averaged across the entire screening population, that is, all women attending screening. The number of cases the AI recalled in the GEMINI study at OP2 was higher than in the prior retrospective study (14.6% versus 13.0%)^11^. This could be due to natural variability in recall rates or an interim change in the mammography machine software version prior to the start of the study. The AI vendor recommended recalibration before the commencement of the study, but this was not feasible because of time and governance constraints. Therefore, a pragmatic approach was taken to use thresholds previously derived from retrospective data from the same site^11^. To ensure that AI performance remains consistent after changes to the imaging systems, AI monitoring and quality assurance methods should be considered, which could include realistic breast phantoms^12^.

Future studies would be strengthened by evaluating several AI algorithms across multiple sites, exploring AI–human interactions according to reader characteristics such as experience^13^, and assessing real-time adaptive AI thresholds. In addition, studies with longer durations and follow-up time would be able to assess the stability of workflow performance over time and whether detecting additional cancers with AI reduces interval cancer rates.

The live AI-Additional Read workflow was run with a higher sensitivity OP (OP2), which enabled simulation with a higher specificity (OP1). This workflow at OP2 flagged a relatively high proportion of cases (12.4%, *n* = 1,345) for additional arbitration. Approximately 90% of these were read faster than, or at the same speed as, a routine mammography examination (an average read opinion takes 59 s^14^), demonstrating that using AI in this way does not increase the overall reading time. Of the 1,345 cases flagged, only 55 were recalled and 11 cancers were diagnosed, resulting in a PPV of 20% for the AI-supported additional human review, similar to routine screening for this center (21.9%). The human readers dismissed over half of the additional arbitrations after reviewing prior mammogram images; the read time for the dismissed cases was less than for those recalled. This suggests that the senior readers performing this additional arbitration were willing and able to critically assess and quickly override the AI opinion, thereby maintaining the expected standards for screening. The simulated AI-Additional Read workflow, at the higher specificity OP, would increase workload less than OP2 (4% versus 6%; *n* = 896 versus 1,345), recall 18% fewer cases (*n* = 45 versus 55) and flag 10 (91%) of the 11 additional cancers detected. Incorporating the ability to consider prior mammograms may improve the functionality of this AI breast screening system and reduce human workload further.

This evaluation of AI for breast screening has identified several ways in which AI could be used in a screening program that optimizes workload savings, CDR and the reduction of false positives while improving, or not compromising, other outcomes. These AI implementation strategies could deliver clinical and operational benefits with trade-offs that can accommodate local requirements and priorities. This is particularly important given the shortfall of radiology consultants and increasing workloads^15^. The technical challenges of integrating AI into clinical practice and setting an appropriate threshold highlight the need for evaluation of AI before implementation, as well as ongoing monitoring. The GEMINI study shows that AI use can be tailored to the needs of clinical sites to improve service delivery.

## Methods

### Sample

This study was considered a service evaluation (NHS Grampian Quality Improvement and Assurance (ID no. 5834)) by the local Research Governance team and did not require ethical approval. The evaluation was registered with the Scottish National Screening Organisation Research and Innovation group and received NHS Grampian Caldicott Approval. Women invited to routine breast screening received an amended invitation letter, providing information about the study, a participant information sheet, a frequently asked question guide and details on how to opt out of the evaluation.

The source population for the GEMINI study included women attending routine breast screening within NHS Grampian, part of the Scottish Breast Screening Service (SBSS), who did not opt out of the study and whose mammograms were read between 27 February and 5 October 2023. SBSS uses the Community Health Index number, a unique patient identifier used in Scotland, to identify women. In the UK NHS Breast Screening Programme, women from 50 to 71 years old are invited triennially for routine mammography screening. A standard mammography examination consisted of mediolateral oblique and craniocaudal full-field digital mammographic views of each breast using a Selenia Dimensions system (Hologic; software v.1.10). All acquisitions followed UK guidelines. Cancers were defined as histologically confirmed cancers present in the breast after surgery (if available) or biopsy.

### The AI system

Mia v.3 (Kheiron Medical Technologies Ltd) is a CE-marked AI system that uses deep learning. Based on a woman’s screening mammograms, it outputs a continuous malignancy prediction value ranging from 0 to 1. The AI indicates that a woman should be invited back for additional examinations if her mammogram’s malignancy prediction value is above a certain decision threshold. The vendor prescribes six pre-set OPs based on different decision thresholds for different configurations. These thresholds are validated using locally acquired data with known outcomes and set before live AI use. During the AI-Additional Read workflow used in the GEMINI study, the AI findings were presented to the arbitration group as a recommended recall opinion with up to three regions of interest, depicted by a yellow outline in each view. The AI system was trained on images from real-world screening programs across different countries and centers, and with equipment from different hardware vendors over more than ten years^16^. However, no data from the GEMINI study NHS Grampian evaluation site was used in the development, training or calibration of the AI system’s machine learning model. The AI OPs used in this study (OP1 to OP4) were tuned and validated locally using a retrospective dataset from the same screening setting^11^.

### Study design

For all women, routine screening practice and standard of care was maintained with all mammograms initially read by two human readers (Fig. 2). Per the center’s routine practice, inexperienced readers were paired with experienced readers (≥3 years of reading experience). In cases of discrepancy, a third human reader made the final clinical decision. Neither the first, second or third human reader (arbitrator) could view the AI opinion.

The AI tool was used live in the clinical workflow with the intention of increasing the CDR (‘AI-Additional Read’ workflow at OP2). If the final clinical decision was not to recall, but the AI tool gave a recall opinion, these cases were additionally arbitrated by 2–3 senior human readers out of a pool of five readers with at least 5 years of experience. During the additional arbitration, the human readers were presented with AI-generated regions of interest. The arbitration panel were instructed to recall women with visible suspicious findings in the breast as well as those with subtle changes that might point to a malignancy, reflecting the UK breast screening program’s interval cancers criteria, recalling at the unsatisfactory or satisfactory with learning points threshold^17^. A study team member (B.T.) recorded the time needed to perform this additional review with a stopwatch. As part of their return consultation, recalled women were met by a clinician who explained that the AI tool had detected a region of interest requiring further evaluation.

The AI opinion for all mammograms was downloaded at the end of the study and provided to the study team in the Grampian Data Safe Haven (DaSH) independent research environment (see ‘Statistics and reproducibility’ section). This design allowed for the simulation of the AI for the ‘triage’ and ‘triage negatives’ workflows. For the ‘triage’ workflow, the AI replaces the second reader when the AI and reader 1 have the same recall/no recall opinion, optimizing workload savings. For the ‘triage negatives’ workflow, the AI replaces the second reader when the AI and reader 1 agree not to recall, reducing workload while avoiding additional recalls. The design also enabled analyses of AI triage in combination with AI as an additional reader (‘AI-Additional Read’) using multiple different OPs to enable the assessment of 17 different workflows.

The primary workflow (specified a priori) combines the live AI workflow (AI-Additional Read at OP2) with the ‘triage negatives’ workflow (at OP3). All workflow definitions, including the choice of the primary workflow, were specified in the Evaluation plan before commencement of data collection.

Further information on study design is available in the Nature Research Reporting Summary linked to this article.

### Data processing

Mammograms from screening attendees with exactly one of each of the four standard views (bilateral (left and right), mediolateral oblique and craniocaudal), identified as female in the Digital Imaging and Communications in Medicine data, were de-identified and sent to Mia’s cloud service, along with the relevant metadata. The Mia server processed cases and returned the Mia results to the Virtual Machine Gateway, where they were returned to the SBSS. Customized Business Objects XI reports containing clinical data, including age, reader opinions, AI opinions and cancer diagnoses, were exported from the SBSS and transferred to the DaSH, a Trusted Research Environment, for analysis.

### Statistics and reproducibility

#### Independence and reproducibility

This study adheres to the STARD-AI reporting statement^18^.

The DaSH team pseudonymized and provisioned the dataset into a secure DaSH workspace, accessible to the NHS Grampian & University of Aberdeen study team only (C.F.d.V., J.A.D., G.L. and L.A.A.). The AI vendor could not access this workspace to ensure that the evaluation was performed independently from the industry partner.

The AI vendor was given access to the data within a separate workspace, where they established the accuracy of the reported results. As this was a real-world evaluation and all participants underwent routine screening, no blinding or randomization was required.

#### Statistical analysis

A Statistical Analysis Plan was created before commencement of data analysis. CDR, recall rate, sensitivity, specificity and PPV were calculated for the AI workflows and routine double reading, with 95% Wilson confidence intervals (CIs). Bootstrapping with 50,000 repetitions was used to generate two-sided 90% CIs for relative differences between the AI workflows and routine double reading for each metric. These CIs were then used to perform non-inferiority tests with a relative margin of 0.1 and an alpha of 0.05, comparing the combination AI workflows with routine double reading for each metric. If non-inferiority was established, a superiority test was executed with an alpha of 0.1. Superiority was established by the lower or upper confidence bound of the ratio being above or below 1, depending on the specific metric. The percentile bootstrap interval was used to estimate the CIs. The relative change in performance for each AI workflow compared to routine double reading, with corresponding 90% CIs, are reported in the Supplementary Table 1. A sensitivity analysis was undertaken for the primary workflow, assuming fewer additional cancers were detected.

All non-inferiority and superiority tests were performed on the lower or upper bound of a CI for the ratio of two metrics. Although the test itself does not involve any assumptions regarding normality and equal variances, the calculation of the CI should be accurate. As the assumption of normality usually does not hold for ratios, a bootstrap procedure was used to calculate these CIs. Data distribution was not assumed to be normal.

A gating strategy was defined for each AI combination workflow for the non-inferiority and superiority testing (Table 5). The order of the tests was defined a priori. No corrections for alpha were applied because the prespecified gating limits the number of hypotheses tested. Furthermore, each combination workflow represents a distinct product configuration that would be deployed independently. If a test was not passed, the next test was exploratory instead of confirmatory.

**Table 5:** Gating strategy for each AI combination workflow

Gating order	Triage negatives + AI-Additional Read						Triage + AI-Additional Read			
	OP3+ OP2	OP3+ OP1	OP4+ OP2	OP4+ OP1	OP2+ OP2	OP2+ OP1	OP2+ OP2	OP2+ OP1	OP1+ OP2	OP1+ OP1
1	CDR	CDR	RR	RR	RR	RR	RR	RR	RR	RR
2	RR	RR	SPEC	SPEC	SPEC	SPEC	SPEC	SPEC	SPEC	SPEC
3	SEN	SEN	SEN	SEN	PPV	PPV	SEN	SEN	SEN	SEN
4	SPEC	SPEC	CDR	CDR	SEN	SEN	CDR	CDR	CDR	CDR
5	PPV	PPV	PPV	PPV	CDR	CDR	PPV	PPV	PPV	PPV

The first OP relates to the triage/triage negatives workflow; the second OP relates to the AI-Additional Read workflow. CDR, cancer detection rate; RR, recall rate; SEN, sensitivity; SPEC, specificity.

The human reader workload was quantified for both the AI workflows and routine double reading, measured as the sum of the number of mammogram examinations read by the first, second and third (arbitration) readers and the number of examinations additionally arbitrated after being flagged by the AI × the number of potential arbiters. The time taken for the additional arbitration was quantified into four categories: 0–30, 30–60, 60–120 and over 120 s. At the center, first and second reads average 30–60 s, with arbitration cases averaging 60–120 s.

Statistical analyses were performed in R (v.4.2.1)^19^. The R library ‘boot’ was used to perform bootstrapping and estimate the CIs^20,21^.

#### Predetermined sample size

The sample size was based on a non-inferiority test for the relative difference between the routine double reading workflow and the primary AI workflow in detecting screen-detected cancers. The agreement rate between the routine double reading workflow and the primary AI workflow was expected to be 95.0%. A percentage of 1.9% of the confirmed positives was expected to only be detected by the routine double reading workflow, while 3.0% was expected to be detected by the AI workflow. Using a one-sided alpha of 0.05 and a non-inferiority margin of 10% relative to the routine double reading workflow proportion, a sample size of 65 confirmed positives was determined before study commencement to have a power of 91.5%. Achieving this sample size ensures that the power for the secondary endpoints for the AI workflow is at least 90%. Because of natural variation in CDR and the time duration between mammography assessment and potential cancer diagnosis, the study conclusion date was estimated to allow 65 confirmed positives to be achieved, resulting in 106 confirmed positives in this sample.

### Reporting summary

Further information on research design is available in the Nature Portfolio Reporting Summary linked to this article.

## Supplementary information

Supplementary Information Supplementary Table 1, Gemini Evaluation and Statistical analysis plans.

Reporting Summary

Supplementary Data Bootstrapped confidence intervals for each AI workflow.

## Source data

Source Data Fig. 3 Source data: workload for each workflow.

## Data Availability

SBSS images and data are not publicly available. Access to the raw, de-identified SBSS data and mammograms is subject to the required approvals by the Data Protection Officer, Caldicott Guardian and other required data agreements being in place. Data access requests are typically reviewed and approved within approximately 6–8 weeks. More information can be found on the DaSH website at www.abdn.ac.uk/research/digital/platforms/safe-haven-dash/. The Evaluation and Statistical Analysis Plans are available in the Supplementary Information. The source data for Fig. 3 has been provided as a Source Data file. Source data are provided with this paper.

## Code availability

The R code used for the statistical analysis can be found at the following GitHub (https://github.com/CFdeVries/gemini-paper) and Zenodo (10.5281/zenodo.14826292)^22^ repositories.
