# Supplementary material for: Prospective evaluation of artificial intelligence integration into breast cancer screening in multiple workflow settings: the GEMINI study
Source: Nat Cancer. 2026 Mar 10;7(3):484–93. doi: 10.1038/s43018-026-01126-1 (PMC13035467; doi:10.1038/s43018-026-01126-1)
Supplement: Supplementary file 1 — Supplementary Table 1, Gemini Evaluation and Statistical analysis plans. [file 43018_2026_1126_MOESM1_ESM.pdf]

nature cancer

Supplementary information

https://doi.org/10.1038/s43018-026-01126-1

Prospective evaluation of artificial intelligence integration into breast cancer screening in multiple workflow settings: the GEMINI study

In the format provided by the authors and unedited

Supplementary Table 1 – Relative change in performance for each AI workflow compared to routine breast screening

	**CDR** (CI)	**Recall rate** (CI)	**Sensitivity** (CI)	**Specificity** (CI)	**PPV** (CI)
**Primary AI Workflow (Triage Negatives + AI-Additional Read)**					
OP3 + OP2	10.4% (5.5 to 16.3)	-0.8% (-4.4 to 2.9)	10.4% (5.5 to 16.3)	0.1% (0.0 to 0.3)	11.3% (5.9 to 17.5)
**Other Combinations of Triage Negatives + AI-Additional Read**					
OP2 + OP1	8.5% (3.5 to 14.4)	-5.6% (-9.1 to -1.9)	8.5% (3.5 to 14.4)	0.3% (0.2 to 0.5)	14.9% (9.2 to 21.4)
OP2 + OP2	9.4% (4.3 to 15.6)	-3.5% (-7.2 to 0.2)	9.4% (4.2 to 15.6)	0.3% (0.1 to 0.4)	13.4% (7.5 to 20)
OP3 + OP1	9.4% (4.7 to 15.1)	-2.9% (-6.2 to 0.6)	9.4% (4.8 to 15.1)	0.2% (0.1% to 0.4%)	12.7% (7.4 to 18.8)
OP4 + OP1	9.4% (4.8 to 15.1)	4.1% (1.3 to 7.1)	9.4% (4.7 to 15.1)	-0.1% (-0.2 to 0.0)	5.1% (0.7 to 10.2)
OP4 + OP2	10.4% (5.5 to 16.3)	6.2% (3.1 to 9.4)	10.4% (5.4 to 16.3)	-0.2% (-0.3 to -0.1)	3.9% (-0.6 to 9.2)
**Combinations of Triage + AI-Additional Read**					
OP1 + OP1	6.6% (1.0 to 12.9)	2.7% (-1.9 to 7.5)	6.6% (1.0 to 12.8)	-0.1% (-0.3 to 0.2)	3.8% (-2.2 to 10.5)
OP1 + OP2	7.5% (1.8 to 14.0)	4.7% (0.0 to 9.7)	7.5% (1.9 to 14.0)	-0.1% (-0.4 to 0.1)	2.7% (-3.4 to 9.4)
OP2 + OP1	8.5% (3.5 to 14.4)	9.9% (5.2 to 15.0)	8.5% (3.5 to 14.4)	-0.4% (-0.6 to -0.2)	-1.3% (-6.8 to 4.8)
OP2 + OP2	9.4% (4.2 to 15.6)	12.0% (7.0 to 17.1)	9.4% (4.2 to 15.6)	-0.5% (-0.7 to -0.2)	-2.3% (-7.8 to 4.0)
**AI-Additional Read Workflows**					
OP1	9.4% (4.8 to 15.2)	9.3% (7.0 to 11.8)	9.4% (4.7 to 15.0)	-0.3% (-0.4 to -0.2)	0.1% (-3.7 to 4.6)
OP2	10.4% (5.4 to 16.3)	11.3% (8.8 to 14.1)	10.4% (5.4 to 16.3)	-0.4% (-0.5 to -0.3)	-0.9% (-5.0 to 3.9)
**Triage Negatives Workflows**					
OP2	-0.9% (-2.8 to 0.0)	-14.8% (-17.5 to -12.2)	-0.9% (-2.8 to 0.0)	0.7% (0.6 to 0.8)	16.3% (12.6 to 20.4)
OP3	0.0% *	-12.2% (-14.7 to -9.8)	0.0% *	0.6% (0.5 to 0.7)	13.8% (10.8 to 17.2)
OP4	0.0% *	-5.2% (-6.9 to -3.5)	0.0% *	0.2% (0.2 to 0.3)	5.4% (3.7 to 7.4)
**Triage Workflows**					
OP1	-0.9% (-4.4 to 2.6)	-5.8% (-9.7 to -1.8)	-0.9% (-4.4 to 2.6)	0.3% (0.1 to 0.4)	5.1% (0.1 to 10.4)
OP2	0.9% (-1.8 to 3.8)	1.4% (-2.6 to 5.7)	0.9% (-1.8 to 3.8)	-0.1% (-0.2 to 0.1)	-0.5% (-5.0 to 4.2)

CDR = Cancer Detection Rate; PPV = Positive Predictive Value; CI = 90% Confidence Interval; OP = Operating point. For the combination workflows, the first OP relates to the Triage/Triage negatives workflow; the second OP relates to the AI-Additional Read workflow.

* Confidence intervals could not be calculated as all values of the statistic (workflow metric/routine screening metric) were equal to 1.

A priori defined tests were performed for all combination AI workflows compared to routine double reading for CDR, recall rate, sensitivity, specificity, and PPV. Test results are indicated with cell colour: =Superior, =Non-Inferior, =Inferior. No cell colour indicates no test was performed.

GEMINI Evaluation Plan

Kheiron Medical Technologies, Grampian Health Board & University of Aberdeen

GEMINI: Grampian's Evaluation of Mia an Innovative National breast screening Initiative

PROJECT CODE	**KMT008**
COMPANY	**KHEIRON MEDICAL TECHNOLOGIES LTD.**<br>Stylus Building, 116 Old Street London,<br>EC1V 9BG United Kingdom
VERSION	**1.0**
DATE	**01 February 2023**

THIS CONFIDENTIAL DOCUMENT IS THE PROPERTY OF KHEIRON MEDICAL TECHNOLOGIES LTD. NO UNPUBLISHED INFORMATION CONTAINED IN THIS DOCUMENT MAY BE DISCLOSED WITHOUT PRIOR WRITTEN APPROVAL OF KHEIRON MEDICAL TECHNOLOGIES LTD.

Signatures

Device Manufacturer: Kheiron Medical Technologies

Print Name	Position	Signature	Date
Dr Annie Ng	Senior Scientist		02/21/2023
Dee Dinneen	Head of UK Implementation and Strategic Partnerships		02/21/2023

Clinical Site: NHS Grampian Health Board

Print Name	Position	Signature	Date
Dr Gerald Lip	Clinical Director, North East of Scotland Breast Screening Programme<br><br>Consultant Radiologist, NHS Grampian		03/07/2023

Independent Evaluation Partner: University of Aberdeen

Print Name	Position	Signature	Date
Professor Lesley Anderson	Chair in Health Data Science, Aberdeen Centre for Health Data Science		03/07/2023

TABLE OF CONTENTS

1 ABBREVIATIONS 5

2 DEFINITIONS 6

3 AIMS AND OBJECTIVES 10
3.1 AIM 10
3.2 OBJECTIVES 10
3.3 PRIMARY OUTCOME 10
3.4 SECONDARY AND EXPLORATORY OUTCOMES 10

4 MEDICAL DEVICE 12
4.1 DEVICE DETAILS 12
4.2 INTENDED USE 12
4.3 DEVICE MANUFACTURER 12

5 EVALUATION DESIGN 13
5.1 SETTING 13
5.2 DESIGN 13
5.3 PROCEDURE 14
5.4 REVIEW OF POSITIVE DISCORDANT CASES 15
5.5 INFORMING PARTICIPANTS 17

6 IMPLEMENTATION 18
6.1.1 Understand 19
6.1.2 Connect 19
6.1.3 Validate 20
6.1.4. Train 20
6.1.5. Launch 20
6.1.6 Monitor 21

7 DATA COLLECTION AND ANALYSIS 22
7.1 METHODS OF DATA COLLECTION 22
7.2 PROPOSED ANALYSIS 22
7.2.1 PRIMARY ENDPOINT 23
7.2.2 SECONDARY AND EXPLORATORY ENDPOINTS 23
7.3 STATISTICAL METHODS 24

8 SAMPLE 24
8.1 ELIGIBILITY CRITERIA 25
8.1.1 INCLUSION CRITERIA 25
8.1.2 EXCLUSION CRITERIA 25
8.2 SAMPLE SIZE 25
8.3 EVALUATION DURATION 25

9 ETHICAL AND REGULATORY CONSIDERATIONS 26
9.1 PPI 26
9.2 DATA MANAGEMENT AND PROTECTION 26

9.3 RISK ASSESSMENT 27

10 TRIPARTITE AGREEMENT 28

11 REFERENCES 28

1 ABBREVIATIONS

CE	Conformitè Europëenne
CC-L	Craniocaudal - Left
CC-R	Craniocaudal - Right
DaSH	Grampian Data Safe Haven
DICOM	Digital Imaging and Communications in Medicine
DPIA	Data Protection Impact Assessment
FFDM	Full Field digital Mammography
GDPR	General Data Protection Regulation
MLO-L	Mediolateral oblique - Left
MLO-R	Mediolateral oblique - Right
NBSS	National Breast Screening System
OP	Operating Point
PACS	Picture Archiving and Communication System
PPI	Patient and Public Involvement
ROI	Region(s) of Interest
SA	Standalone
SR	Silent Reader
SR/XR	Silent Reader/Extra Reader Combination
UUID	Universally Unique Identifier
XR	Extra Reader

2 DEFINITIONS

Configurations & Workflows:

1. **'operating point' (OP)**: a pre-determined decision threshold for the Mia software that specifies a tradeoff between sensitivity and specificity.

2. **'standalone' (SA)**: device's case-wise malignancy assessment independent of any workflow or reader opinion on cases it has read. In this evaluation, Mia standalone performance will be assessed at operating points A1, A2, A3, and A4.

3. **'discordance arbitration':** arbitration of cases where two or more readers disagree in order to make a final double reading decision according to local standard operating practice.

4. **'consensus arbitration':** arbitration of cases where two or more readers agree to recall in order to make a final double reading decision according to local standard operating practice.

5. **'double reading' (DR):** a double reading workflow where two readers (R1, R2) read every case. When R1 and R2 agree to not recall, the case is not recalled. When R1 and R2 disagree, discordance arbitration is conducted to make the final double reading decision. When R1 and R2 agree to recall, arbitration may also be conducted to make the final double reading decision, depending on the site's arbitration practice in what is called 'consensus arbitration'. See Figure 1.

6. **'double reading with Mia':** a double reading workflow that incorporates Mia as an independent reader.

7. **'independent reader':** configuration where Mia serves as an independent reader. Potential workflows are detailed below:

    a. **'Extra reader' (XR):** a double reading workflow utilising Mia to be an independent reader in addition to double reading. In double reading workflows, Mia's additional read serves as a third independent read. 'Positive discordant' cases (see definition in 'samples' sections) are then subsequently reviewed by a human reader or group of human readers depending on local practice in an 'extra arbitration' step where the case is additionally reviewed. During 'extra arbitration', readers will have access to the AI output, prior arbitration decision and prior mammograms/clinical notes, and will review the case with the aim of detecting additional cancers. This workflow will primarily use Mia operating point A2 in the evaluation but results will also be generated for operating point A1 via simulation. Results for operating point A1 are expected to be conclusive as the positive discordant cases resulting from Mia operating point A1 will be a subset of those resulting from A2, i.e. no censorship should occur from the evaluation process.

    b. **'double reader triage' (nDRT):** a double reading workflow utilising Mia to be an independent reader in a portion of cases. Mia serves as an independent second reader in cases where Mia and the first reader (R1) agree to not recall. Otherwise, a second human reader (R2) serves as the second reader. Double reading is carried out using R1, either Mia or R2, and arbitration when required. This workflow is sometimes referred to as 'non-interactive double reader triage' (nDRT) or 'non-interactive partial independent reader' (nPIR). This workflow is analysed in this evaluation using Mia operating points A2, A3, and A4 at the end of the core project time period.

c. **‘independent reader’ (nIR):** a double reading workflow that uses the first human reader (R1) to be the first reader. Mia is used as the second reader if R1 and Mia agree. When R1 and Mia disagree, the second reader (R2) is used as the second reader; upon disagreement between first and second reads, arbitration is conducted to determine the final decision. In the case that consensus arbitration is followed at a site, arbitration is also conducted when first and second reads agree to recall. Essentially, Mia is used as the second reader when R1 and Mia agree. If they disagree, Mia’s opinion is disregarded and the Reader 2 (R2) decision is used, thus the workflow defaults to standard double reading. This workflow is sometimes referred to as ‘non-interactive independent reader’ (nIR). This workflow is simulated in this evaluation using Mia operating points A1 and A2. The simulation will be an approximation because there may be cases suggested to be recalled by this workflow which are not recalled by the standard double reading or extra reader workflows (expected 0.3% of all screens). This subset of suggested recall cases will not be recalled to maintain the standard of care double reading at the evaluation site and to minimise impact on women in the evaluation. This subset of suggested recall cases will be taken into account when measuring metrics such as recall rate, however, since they will not actually be recalled, it will not be possible to determine the number of cancers detected in the subset. Metrics such as cancer detection rate will therefore be a lower bound estimate.

d. **‘combination workflow’:** a combination of nDRT or nIR workflows with the XR workflow. For example:

   i. nDRT+XR is a combination of nDRT and XR workflows. See Figure 1.
   ii. nIR+XR is a combination of nIR and XR workflows

   Considering the varying operating points (OP) that each workflow can use, the following is a full list of combination workflows that will be evaluated:

   i. nDRT(A3)+XR(A2) - double reader triage at OP A3 and extra reader at OP A2 is the **‘primary workflow’** evaluated in this evaluation.
   ii. nDRT(A3)+XR(A1) - analysed at the end of the core project time period
   iii. nDRT(A2)+XR(A2) - analysed at the end of the core project time period
   iv. nDRT(A2)+XR(A1) - analysed at the end of the core project time period
   v. nDRT(A4)+XR(A2) - analysed at the end of the core project time period
   vi. nDRT(A4)+XR(A1) - analysed at the end of the core project time period
   vii. nIR(A2)+XR(A2) - approx. simulated at end of the core project time period
   viii. nIR(A2)+XR(A1) - approx. simulated at end of the core project time period
   ix. nIR(A1)+XR(A2) - approx. simulated at end of the core project time period
   x. nIR(A1)+XR(A1) - approx. simulated at end of the core project time period

Note: Extra Reader is the only workflow listed above which will impact patients in this evaluation as additional reviews will provide the opportunity for additional cancers to be found. All other mentioned workflows above will not be implemented in the service, however, their potential impact will be analysed or approximately simulated during analyses based on result outputs from the project.

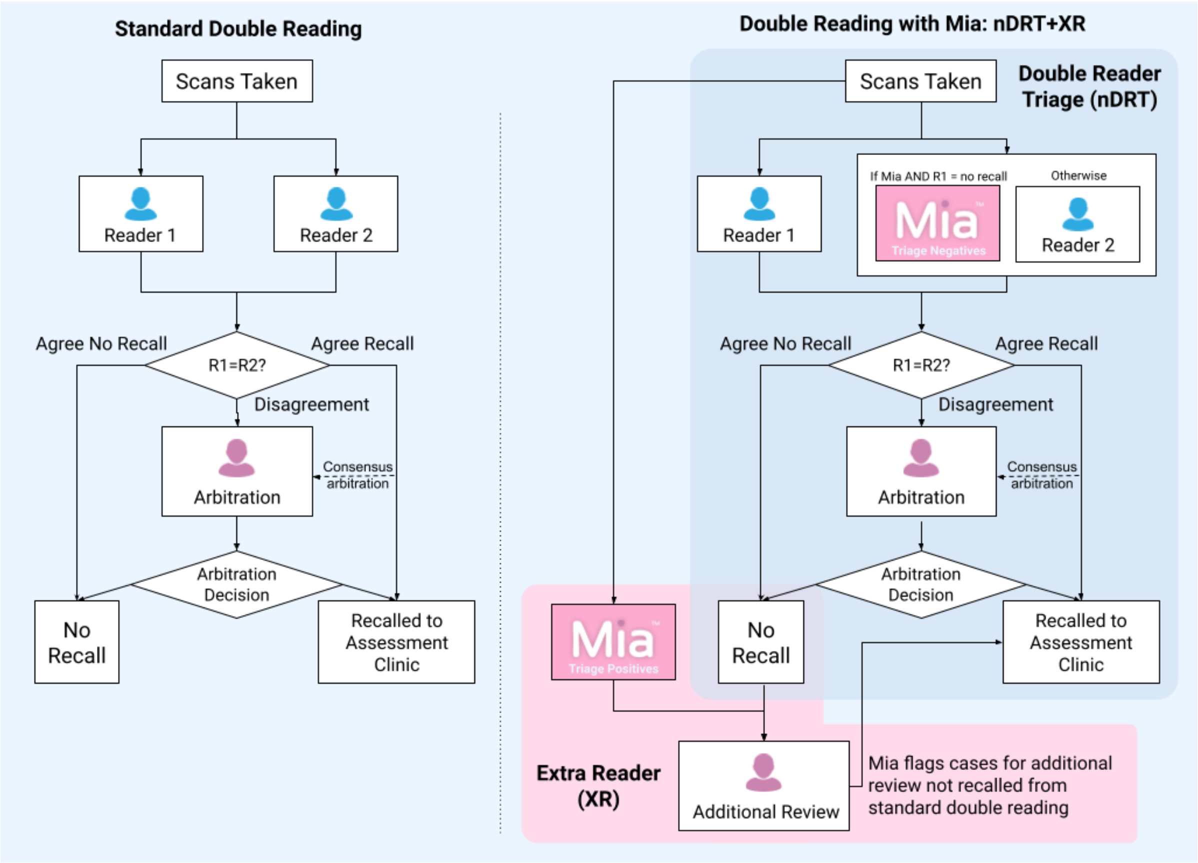

Figure 1: Standard double reading (left) and combination workflow nDRT+XR (right). A combination of double reader triage (nDRT) at OP A3 and extra reader (XR) at OP A2 is the primary workflow evaluated in this project, which will be compared to standard double reading.

Samples:

1. **'total number of cases':** total number of sampled cases relevant for an endpoint (varies by endpoint).
2. **'positive discordant cases':** cases where Mia (SA) suggests to recall and the double reading (DR) opinion is to not recall.
3. **'negative discordant cases':** cases where Mia (SA) suggests to not recall and the double reading (DR) opinion is to recall.
4. **'confirmed Positive (CP) cases':** cases where a malignancy is confirmed by a decisive biopsy, cytology or histology of the surgical specimen downstream within a follow-up period (expected c.2-3 months).
5. **'confirmed Negative (CN) cases':** cases where a negative follow-up result is available which includes mammography reading beyond a time period based on the screening interval (expected c.34 months) without sign of malignancy between.
6. **'non-positive cases' (NP):** cases where a malignancy is not confirmed within a 'positive evidence collection period'.
7. **'core project time period'**: time period between the first and last case recruited into the

evaluation.

8. **'positive evidence collection period'**: period after the last case recruited into the evaluation i.e. the core project time period, where evidence of malignancy and cancer subtype information will be collected for cases (circa 2-3 months).

Note: A Statistical Analysis Plan (SAP) will be created confirming time period details used in definitions and handling of missing data.

Metrics:

1. **'arbitration rate' (AR):** the number of cases arbitrated (either by discordance or consensus arbitration) divided by the total number of cases.
2. **'positive discordance rate':** the number of positive discordant cases divided by the total number of cases.
3. **'negative discordance rate':** the number of negative discordant cases divided by the total number of cases.
4. **'recall rate' (RR):** the number of cases recalled divided by the total number of cases.
5. **'recall rate on positive discordant cases' (RRpd):** the number of positive discordant cases recalled divided by the total number of positive discordant cases.
6. **'negative flag rate' (NFR):** metric associated with the nDRT workflow; the number of cases 'negative flagged' (i.e. not deemed requiring double reading) divided by the total number of cases.
7. **'cancer detection rate' (CDR):** the number of positive cases recalled divided by the total number of cases.
8. **'sensitivity' (SEN):** the number of positive cases recalled divided by the total number of positive cases.
9. **'specificity' (SPEC):** the number of confirmed negative (CN) cases not recalled divided by the total number of confirmed negative (CN) cases.
10. **'Specificity on non-positives' (SPECnp):** the number of non-positive cases not recalled divided by the total number of non-positive cases.
11. **'positive predictive value' (PPV):** the number of positive cases recalled divided by the total number of cases recalled.
12. **'negative predictive value' (NPV)**: the number of confirmed negative (CN) cases not recalled divided by the total number of cases not recalled.

3 AIMS AND OBJECTIVES

3.1 AIM

To evaluate the safety and efficacy that Mia can bring to the breast cancer screening workflow through opportunities of clinical and operational benefit.

3.2 OBJECTIVES

- Deploy Mia in the extra reader (XR) workflow within the breast screening service.
- Silently run Mia in double reading workflows to retrospectively analyse their potential impact and contribution to the breast screening service.
- Collect data from Mia, the Scottish Breast Screening System (SBSS), and screening mammograms to analyse key clinical outcomes in breast screening including possible workload savings in breast screening (see Sections 3.3 and 3.4 for summaries of planned outcomes).

3.3 PRIMARY OUTCOME

A non-inferiority test (and estimates of the associated absolute and relative differences) will be performed for cancer detection rate (CDR) between:

- standard double reading (without Mia), and
- the primary double reading workflow with Mia being evaluated in this evaluation i.e. a combination of 'double reader triage' (nDRT) using Mia at OP A3 and 'extra reader' (XR) using Mia at OP A2 (nDRT(A3)+XR(A2)).

3.4 SECONDARY AND EXPLORATORY OUTCOMES

Note: Full detail of secondary endpoints can be found in Section 7.2.2. Examples of relevant ***clinical metrics*** *include recall rate (RR), cancer detection rate (CDR), sensitivity (SEN), specificity (SPEC), and positive predictive value (PPV).* ***Double reading without Mia*** *is standard double reading.* ***Double reading with Mia*** *includes various combinations of 'independent reader' workflows (and their components) at varying operating points. Standalone performance of Mia also involves varying operating points. A breakdown of the basis for which non-eligible cases were excluded will be assessed. A review of the reasoning behind extra reader arbitration opinions will also be evaluated.*

1. Measurement of clinically relevant metrics for double reading with Mia (i.e. 'independent reader workflows) and without Mia (i.e. standard double reading) to quantify the performance of double reading with and without Mia.

2. Measurement of workload impact in terms of R2 and arbitration reads and overall workload impact for double reading with Mia compared to without Mia.

3. Measurement of relevant clinical metrics (see note above) for each reader (R1, R2 and Mia Standalone) to understand their contribution to double reading.

4. Comparisons between double reading with Mia (i.e. 'independent reader workflows) and without Mia (i.e. standard double reading) to understand differences in their performance and the spectrum of disease they detect by assessing:
    a. Non-inferiority and superiority tests and measurement of absolute and relative differences on clinically relevant metrics,
    b. Descriptive statistics of cancer subtypes picked up.

5. Subgroup analyses for double reading with Mia and without Mia and for each reader (R1, R2 and Mia Standalone) to understand their performance on specific subgroups (these are exploratory analyses).

4 MEDICAL DEVICE

4.1 DEVICE DETAILS

Mia™ is a CE marked medical device indicated to aid readers in the interpretation of breast imaging examinations for the early detection and diagnosis of breast cancer. The only input Mia uses to read a 2D full-field digital mammogram (FFDM) case is the four view images of the FFDM: MLO-R, CC-R, MLO-L, CC-L. Mia can provide binary recall decisions for cases at six operating points (OP): A1, A2, A3, A4, A5, and A6, which are relevant for various workflows.

In general, OP 1-3 are relevant for independent reader type workflows while OP 4-6 are relevant for triage type workflows. Operating points A1, A2, A3, and A4 are relevant to the workflows involved in this evaluation. Double reader triage at OP A3 and extra reader at OP A2 are the most relevant for the primary workflow in this evaluation.

Cases ‘read’ by Mia are considered cases eligible for Mia to read in this evaluation for which Mia has provided a case-wise recall suggestion for. Cases ‘analysed’ by Mia are case's that may have been processed by Mia to determine a recall decision, but are not considered eligible for the evaluation and therefore ignored in analysis. Cases can also be presented to Mia but excluded from analysis or reading automatically by Mia based on certain exclusion criteria e.g. males.

4.2 INTENDED USE

The device is indicated to aid readers in the interpretation of breast imaging examinations for the early detection and diagnosis of breast cancer. The device can serve as a concurrent reader in all reading workflows, including single and/or double reading workflows. Additionally, the device can serve as an independent second or third reader in blinded or unblinded workflows. The device can also be used in the triaging of examinations for workflow prioritisation, and resource management. Output of the device may include classification information such as case-wise suggestion (suspicion of malignancy and/or recommendation that the subject/patient ‘should be recalled’ or ‘should not be recalled’ for further assessment), and localisation information of suspected malignancies such as left and/or right side, projection (e.g. CC and/or MLO views), and region(s) of interest (ROI).

4.3 DEVICE MANUFACTURER

Kheiron Medical Technologies Ltd
Stylus Building
116 Old Street London,
EC1V 9BG, United Kingdom

5 EVALUATION DESIGN

5.1 SETTING

This evaluation will take place within the North East of Scotland Breast Screening Programme within NHS Grampian Health Board. Participants will be women who attend the breast screening sites for mammography screening during the time frame of the evaluation, based on the below inclusion and exclusion criteria outlined in Section 8.1. Only NHS Grampian clients will be included.

5.2 DESIGN

This project will evaluate the safety and efficacy of the use of Mia, an Artificial Intelligence (AI) software medical device, in double reading for breast cancer screening compared to standard double reading without Mia.

The evaluation will evaluate the use of Mia for two main purposes:

1. 'Triage Negatives' to provide workload savings
2. 'Triage Positives' to provide opportunities for increased cancer detection.

The primary double reading workflow with Mia to be evaluated is a combination of two workflows, 'double reader triage' and 'extra reader', which serve to 'triage negatives' and 'triage positives', respectively. **The primary outcome will evaluate the combined workflow of 'Mia double reader triage' at operating point A3 and 'Mia extra reader' at operating point A2 ('primary workflow').**

The first part of the workflow, to **'triage negatives'**, will have **no impact** on the service as the workflow will be exactly simulated during retrospective analysis. The service can conduct standard double reading according to their routine clinical practice during the evaluation.

The second part of the workflow, to **'triage positives'**, occurs after the service's full double reading workflow is performed with their standard arbitration practice. In this part of the workflow, Mia is used as an **extra reader**, flagging cases where Mia suggests to recall which double reading did not suggest to recall (termed 'positive discordant' cases). These flagged positive discordant cases will undergo additional review by experienced readers within the service with access to the AI output, prior arbitration decision and prior mammograms/clinical notes. If the readers determine that the AI has detected a case that requires further evaluation, the client will be recalled and offered a follow-up appointment, providing an opportunity to find more cancers.

Variants of the primary workflow will also be analysed at various operating point combinations. Further, each of the workflow components will also be analysed independently at relevant operating points to understand their impact and contribution to the combined workflows. Table 1 provides additional information on the various workflow components. Further information on operating points can be found in Sections 2 and 4.1.

Table 1: Additional information on the various workflow components.

Use and purpose of Mia	Workflow and Operating Point (OP) Variations	Changes to the screening workflow in the study
Triage Negatives for workload savings and sustainability	**Double reader triage (nDRT)**<br>- analysed at OP A3 for primary workflow<br>- also analysed at OPs A2 and A4<br>**Independent reader (nIR)***<br>- approximately simulated at OPs A1 and A2	**No impact:** Mia will work silently in the background during standard double reading, and these workflows will be **analysed** or **simulated** based on standard double reading. This means potential workload savings will be measured, but not actually saved in the study at the study site. This is

		to enable the paired study design.
Triage Positives for increasing cancer detection	**Extra reader (XR)**<br>- OP A2 will be used at the study site in the study for the primary workflow<br>- Results for OP A1 will also be analysed based on results from OP A2	**Study site will conduct additional review** of about 1/10th of cases to potentially find more cancers. A small number of women (estimated to be about 38 i.e. 0.37%) will be recalled and offered follow-up appointments as a result of the additional reviews.<br><br>Women recalled from an additional review will receive an additional phone call and letter from the project team. There may be a short delay in women receiving their results, but NHS Grampian expects to contact women within three weeks of their appointment.

* nIR triages positives as well as negatives to provide workload savings.

Performance in double reading with Mia will be compared to standard double reading without Mia to ensure performance is at the least maintained, while providing workload savings and potentially additional clinical benefits such as increased cancer detection rate (during the evaluation time frame) and reduced recall rate (to be evaluated retrospectively). Mia's standalone performance will be assessed to characterise Mia's contribution as an independent reader to various double reading workflows. Mia's performance will be assessed in terms of relevant metrics such as recall rate (RR), cancer detection rate (CDR), sensitivity (SEN), specificity (SPEC), and positive predictive value (PPV).

5.3 PROCEDURE

The implemented workflow (Figure 2) will consist of standard double reading, with Mia processing mammograms silently in the background, followed by the use of Mia in the Extra Reader (XR) workflow to flag additional clients for review.

This workflow includes the following steps (step numbers match the circled numbers in Figure 2):

1. All eligible client's mammograms will continue to be read according to the service's routine standard double reading workflow. Therefore, Reader 1 (R1) and Reader 2 (R2) will read all mammograms as normal.

2. In addition, Mia will read all eligible client cases. Human Readers R1 and R2 will always be blinded to Mia's opinion. Clients that are not eligible (based on opt out, inclusion and exclusion criteria, technical limitations) and can be automatically identified to not be analysed by Mia will not be submitted for reading by Mia. Ineligible clients that cannot be automatically identified for exclusion may be analysed by Mia but will be excluded from results calculations as appropriate.

3. Opinions for R1 and R2 will be compared for all clients to determine which cases require arbitration as normal.

4. Standard double reading arbitration will be conducted according to the service's local standard operating practice.

5. Any clients recalled from the standard double reading workflow will continue to be recalled

as per the service's standard operating procedures (SOPs). There may be a short delay (up to 72 hours) in women receiving their results to accommodate the extra time required for additional reviews of mammograms in step 6. But NHS Grampian expects to contact women within three weeks of their appointment. Any delay would not impact the clinical care of women.

6. After standard double reading, Mia will flag positive discordant cases (where Mia suggests to recall which standard double reading suggests to not recall) for additional human review. Some clients may be recalled from this additional review to give the opportunity to find further cancers. These women will receive a phone call and a letter informing them of the suggested recall from the service evaluation.

7. Outside of standard double reading, Mia will read mammograms silently in the background. This information will be used during later analyses, with no impact on clinical care, to analyse or simulate workflows.

Additionally, follow-up positive evidence (circa 2-3 months) about clients will be collected after the last mammogram read by Mia to determine whether cancers were detected in the women sent for further assessment.

Client demographic information, reader opinions, and data on the case history, procedures and outcomes (such as cancer information) will be collected from the Scottish Breast Screening System (SBSS) to support analysis, interpretation of results, truthing, understanding of the sample of women and subgroup analyses. Data required for analysis of the evaluation such as data collected from SBSS and Mia decision outputs will be anonymised and moved into the Grampian Data Safe Haven (DaSH).

5.4 REVIEW OF POSITIVE DISCORDANT CASES

A clinical SOPs will include details of how discordant case reviews will be conducted during the initial implementation of Mia phase of the project (See Section 6) and during the evaluation.

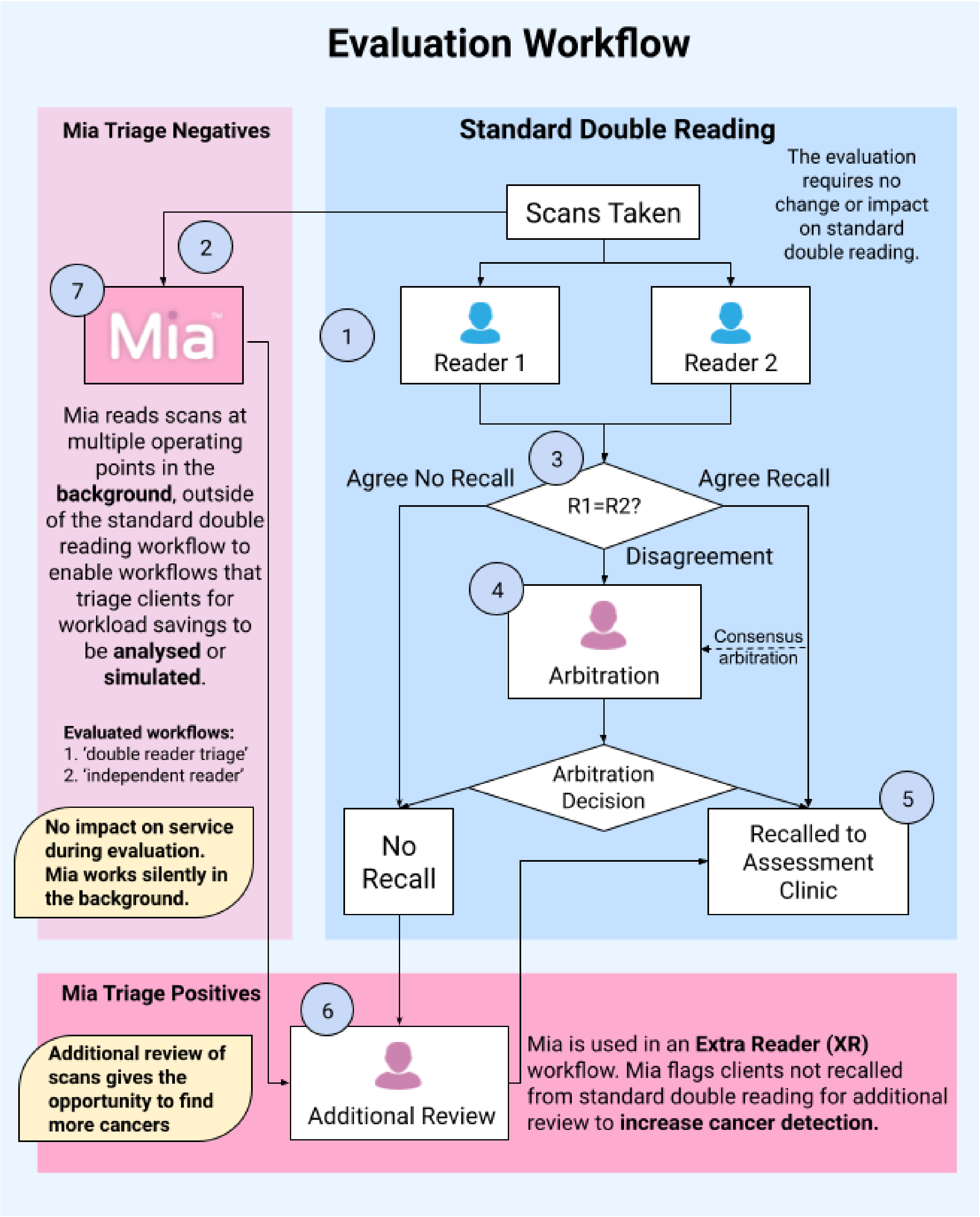

Figure 2: Workflow diagram showing the flow of mammograms and the involvement of Mia, including the simulation of workflows for the Triage of Negatives, which has no impact on standard double reading, and the flagging of clients for additional review for the Triage of Positives which supports additional cancer detection after standard double reading. Points 1-7 are described in Section 5.3.

5.5 INFORMING PARTICIPANTS

In the evaluation of the service, the sample obtained will be representative of a real-world screening population enabling robust evaluation of the impact and benefits that Mia may have in real-world deployments in double reading. Informed consent is not required for a service evaluation. However, the project will use an opt out approach to ensure women are informed and have a choice as to whether they are included in the evaluation taking place at the time of their appointment.

Women can opt-out of the study up until their mammogram is taken. There will be multiple opportunities provided for women to learn about the project and to opt out up to that point:

a. A participant information sheet (PIS) will be included with the standard invitation letters for the local breast screening programme to provide full information on the project and how to opt out. This will provide details for the project website where women can read the Frequently Asked Questions (FAQs) information should they have further queries.
b. Information posters will be placed around the breast screening clinic providing details about the project and the opt out procedure.
c. Copies of the Frequently Asked Questions (FAQs) sheet will be printed and available around the clinic and will also be on hand for staff to use to help answer questions. Training will also be provided for all staff before project commencement. Verbal information will be provided on the project as part of the 'check in' process with staff at reception and/or radiographer, with a reminder that there is an opportunity to opt out. Clients will be able to ask staff any questions they may have.

Client-facing materials have been developed in collaboration with, and reviewed by, the established Kheiron Patient and Public Involvement (PPI) Advisory Board to ensure ease of understanding and use of appropriate language. These include a participant information sheet, a list of FAQs and a project website. Project materials have also been reviewed by the local Grampian PPI representatives, including the Aberdeen Centre Health Data Science PPI group, to ensure local women have had input into their development.

The Clinical Lead has overall responsibility for opt out procedures at the site and to ensure that any member of staff who is delegated responsibility within the opt out procedure is appropriately trained and acts in accordance with this proposal.

Clients who withdraw will be asked for their reason for withdrawal, however the right of a client to refuse participation without giving reasons will be respected and they will continue to receive standard of care double reading.

It is only possible for a client to opt out of having their mammogram read by Mia up until their mammogram is taken. After this point, it is not possible for a client to withdraw their data.

6 IMPLEMENTATION

Prior to the start of the evaluation, Mia will be implemented at the evaluation site according to six stages, which are detailed in sections 6.1.1 to 6.1.6.

Key responsibilities of each party are also included in the project contract.

Figure 3 demonstrates the data flows during the GEMINI project.

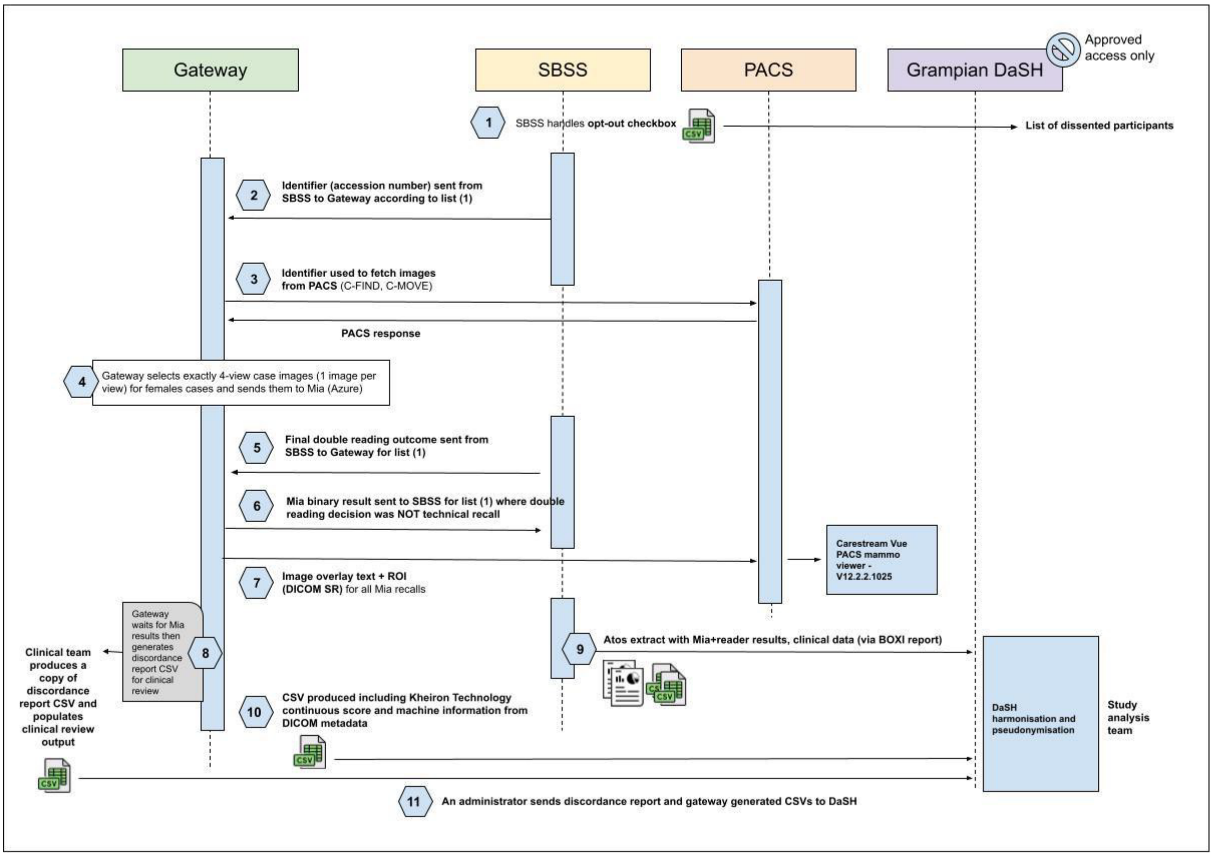

Figure 3: Data flows during GEMINI project

The steps referred to in Figure 3 are as follows:

1. Client opt outs are handled in SBSS using the opt out checkbox field. This generates a list of clients for use within the evaluation, and a list of dissented clients.
2. A list of identifiers, generated using the Accession Number, is sent from SBSS to the Gateway for the list of clients for use in the evaluation from Step 1.
3. The Gateway uses this list of identifiers to fetch the corresponding client images from PACS.
4. Cases with exactly four views (MLO-L, MLO-R, CC-L, CC-R) and identified as female in the DICOM data are de-identified and sent, along with necessary metadata information, to Mia's cloud service. The Mia server processes cases and returns the Mia results to the Gateway.
5. SBSS sends the human double reading final outcome result to the Gateway.
6. The Gateway returns a binary result to SBSS for all eligible cases.

7. For recall cases, Mia’s results are made available to the clinical team in PACS.
8. SBSS final outcome results are associated with the Mia results. Once all necessary data is available, the Gateway generates a discordance report CSV for clinical review. The clinical team produces a copy of the discordance report CSV and populates it with their clinical review output.
9. Customised BOXI reports will be exported from SBSS by the screening team to Grampian’s Data Safe Haven (DaSH). Only approved members of the project team will have access to de-identified data inside DaSH.
10. A CSV is produced in gateway which includes the continuous score generated by the Kheiron Technology and machine information from DICOM metadata. Note: the continuous score is not a standard output of the Mia medical device.
11. An administrator sends the populated discordance report and gateway generated CSVs to DaSH.

6.1.1 Understand

Scope and **understand your needs** and **local set up**, to **tailor** this implementation and project

Activities	Outputs	Step Acceptance Criteria
Kick-off session and planning with all key stakeholders	Detailed plan and milestones	Mutually agreed plan
Detailed scoping of technology systems and clinical workflows	Agreed ways of working including roles and responsibilities, reporting, meetings	Signed off site survey and data privacy agreement
Confirm approvals required on the route to go-live	Site survey (technical specs)	
Identify project impact goals and success metrics	Technical & clinical teams engaged	

6.1.2 Connect

Connect Mia to local infrastructure

Activities	Outputs	Step Acceptance Criteria
Install Kheiron Gateway	Working and tested integration	Evidence of satisfying test criteria and signed-off test report
Set up Mia Azure connection		
Set up PACS connection		
Set up clinical data connection		
Configure within workflows		
Test interface end-to-end		
Configure ready to move from test to live environment		

6.1.3 Validate

Check Mia's performance with local data & population.

Activities	Outputs	Step Acceptance Criteria
Technical prep (virtual machine setup with PACS endpoint)<br><br>Data collection<br><br>De-identification & site approval<br><br>Data transfer to Kheiron's cloud<br><br>Analysis of data by Mia<br><br>Performance evaluation against ground truth to establish optimum recall/no recall threshold. Potential option to calibrate, depending on performance	Validate Report: outlining results of the performance evaluation	Confirmed generalisability of Mia to local data - performance at Kheiron's expected levels

A validate report will be provided before the GEMINI evaluation period can commence.

6.1.4. Train

Train clinical and administrative teams in the use of Mia, in preparation for launch

Activities	Outputs	Step Acceptance Criteria
Separate product training for clinical and administrative users, based on day-to-day Mia usage.	All required team members attend training and where necessary demonstrate correct usage.<br><br>Reference materials provided.	Product training completed by all necessary members of staff.

6.1.5. Launch

Preparations for launch, including final approvals. Then go live.

Activities	Outputs	Step Acceptance Criteria
Communications planning and execution for site's team and patients (supported by Kheiron, done by partner site)<br><br>Change clinical, technical and operational processes where required<br><br>Clinical, business and other approvals<br><br>Agree mutual monitoring process<br><br>Kheiron Helpdesk on-boarding<br><br>Go-live	Live usage on prospective cases.	Launch approved (based on list of approvals defined in the Understand phase)

6.1.6 Monitor

Monitor Mia's performance post-launch and ensure expected results are being met

Activities	Outputs	Step Acceptance Criteria
Proactive monitoring of performance post-launch<br><br>Transition to post-launch support system and able to raise tickets<br><br>Confirm long term expectations and value expected, and mutually plan	Correct usage of Mia after go-live<br><br>Continued performance at expected levels<br><br>Site is able to independently use Mia and review results<br><br>Joint success plan	Project completion once support handover & continued performance is verified.

7 DATA COLLECTION AND ANALYSIS

7.1 METHODS OF DATA COLLECTION

Source data collection will be from SBSS records, imaging data and the AI software. Further details regarding data handling and data flows (including data required for the evaluation) are detailed in a separate data protection impact assessment (DPIA) and other supporting documentation.

7.2 PROPOSED ANALYSIS

Standalone (SA) analyses of Mia will use Mia operating points A1-4.

Mia XR will be used at operating point (OP) A2 in the study workflow.

The following double reading workflows with Mia will be simulated:

1. Double reader triage (nDRT):
    a. nDRT(A3)
    b. nDRT(A2)
    c. nDRT(A4)
2. Independent reader (nIR):
    a. nIR(A2)
    b. nIR(A1)
3. Extra reader (XR):
    a. XR(A2)
    b. XR(A1)
4. Combination workflows:
    a. nDRT(A3)+XR(A2)
    b. nDRT(A3)+XR(A1)
    c. nDRT(A2)+XR(A2)
    d. nDRT(A2)+XR(A1)
    e. nDRT(A4)+XR(A2)
    f. nDRT(A4)+XR(A1)
    g. nIR(A2)+XR(A2)
    h. nIR(A2)+XR(A1)
    i. nIR(A1)+XR(A2)
    j. nIR(A1)+XR(A1)

The following references to the workflows will be used when describing endpoints:

- ‘Double reading with Mia (nDRT)’ refers to 1a-c above.
- ‘Double reading with Mia (nIR)’ refers to 2a-b above.
- ‘Double reading with Mia (XR)’ refers to 3a-b above.
- ‘Double reading with Mia (nDRT+XR, nIR+XR)’ refers to 4a-j above.
- ‘Double reading with Mia (nDRT+XR)’ refers to 4a-f above.
- ‘Double reading with Mia (nIR+XR)’ refers to 4g-j above.
- ‘Double reading with Mia (nDRT, nIR)’ refers to 1-2 above.
- ‘Double reading with Mia (nDRT, nIR, XR)’ refers to 1-3 above.
- ‘Double reading with Mia (XR, nDRT+XR, nIR+XR)’ refers to 3-4 above.
- ‘Double reading with Mia (nDRT, nIR, XR, nDRT+XR, nIR+XR)’ refers to all possible double reading workflows with Mia 1-4 above.

Descriptive analyses on cancer subtype will include the following variables:

- invasivity i.e. invasive vs non-invasive
- receptor status
- for invasive cancers:
  - tumour size based on imaging
  - lymph node status
  - histological grade
  - tumour type e.g. invasive ductal carcinoma (IDC) and invasive lobular carcinoma (ILC)

Further subgroup analyses will be done on age, breast density, and prevalent (first-timer screens) vs incident (returning participant screens) status. Further analysis on concordant and discordant cases between Mia and human reader(s) will also be done to understand how Mia and human readers can optimally be combined in collaboration to maximise clinical outcomes for participants.

A breakdown of the basis for which non-eligible cases were excluded will be assessed.

A review of the reasoning behind extra reader arbitration opinions will also be evaluated.
The following database locks will take place:

1. Database lock 1: during the core project time period for interim analyses; any available positive evidence and cancer subtype information available will be included;
2. Database lock 2: after the core project time period; analyses for the full core project time period not requiring positive evidence or cancer subtype information will take place after this database lock;
3. Database lock 3: after the positive evidence collection time period; remaining analyses requiring positive evidence and cancer subtype information will take place after the positive evidence collection time period database lock.

Separate, additional database locks may take place for the collection of data not available to be collected at database lock 1, 2, or 3, followed by relevant analyses.

7.2.1 PRIMARY ENDPOINT

At the end of the positive evidence collection time period, a non-inferiority test (and estimates of the associated absolute and relative differences) will be performed for cancer detection rate (CDR) between:

- standard double reading (without Mia), and
- the primary double reading workflow with Mia being evaluated in this evaluation i.e. a combination of ‘double reader triage’ (nDRT) using Mia at OP A3 and ‘extra reader’ (XR) using Mia at OP A2 (nDRT(A3)+XR(A2)).

7.2.2 SECONDARY AND EXPLORATORY ENDPOINTS

1. For double reading with Mia (nDRT, nIR, XR, nDRT+XR, nIR+XR) and without Mia, measure:
   a. Recall Rate (RR), Cancer Detection Rate (CDR), Sensitivity (SEN), Positive Predictive Value (PPV), Specificity on non-positives (SPECnp) per workflow and absolute and relative differences between with and without Mia workflows
   b. SEN of:
      i. Cancers detected by both double reading with Mia and without Mia (i.e. intersection of cancers detected by both workflows)
      ii. Cancers detected by double reading with Mia only (and not double reading without Mia)
      iii. Cancers detected by double reading without Mia only (and not double reading with Mia)

   c. Descriptives of cancer subtypes per workflow and among agreed and discordant cancers between compared workflows
   d. Subgroup analyses
2. Non-inferiority and superiority tests and associated absolute and relative differences of AR, RR, CDR, SEN, PPV, SPECnp for:
   a. Double reading with Mia (nDRT+XR) compare to without Mia (excluding primary endpoint)
   b. Double reading with Mia (nIR+XR) compare to without Mia
3. For double reading with Mia (nDRT, nIR, nDRT+XR, nIR+XR), measure:
   a. AR
   b. Workload savings compared to double reading without Mia:
      i. Reduction in the number and proportion of cases required to be read by R2 (i.e. negative flag rate (NFR) for nDRT)
      ii. Reduction in the number and proportion of cases required to be read by Arbitration
      iii. Overall workload savings if arbitration read time takes 1x, 2x, 3x, or 4x first or second reads
4. For double reading with Mia (XR, nDRT+XR, nIR+XR), measure:
   a. Positive discordance rate, negative discordance rate, RRpd (on positive discordant cases) (note: RRpd for XR is absolute difference of RR compared to double reading without Mia)
   b. Descriptives of cancer subtypes on positive discordant cases per workflow and among agreed and discordant cancers when compared to double reading without Mia
5. For single readers R1, R2, and Mia Standalone, measure:
   a. RR, CDR, SEN, PPV, SPECnp per single reader and absolute and relative differences between human readers (R1, R2) and Mia
   b. SEN of:
      i. Cancers detected by both R1 and Mia (i.e. intersection of cancers detected by both R1 and Mia),
      ii. Cancers detected by R1 only (and not Mia), and
      iii. Cancers detected by Mia only (and not R1)
   c. Descriptives of cancer subtypes per single reader and among agreed and discordant cancers between single readers
   d. Subgroup analyses (these are exploratory analyses)

7.3 STATISTICAL METHODS

Point estimates plus 90% and 95% confidence intervals will be provided for all relevant primary and secondary endpoint variables.

For non-inferiority tests for all metrics, one-sided 95% CIs and a 10% relative difference margin will be used. Wherever any non-inferiority tests pass, a superiority test with 90% CIs will be performed. Confidence interval estimation for the difference of metrics will account for the paired nature of the data.

An interim analysis will be conducted during the evaluation (core project time period) including measurements of relevant clinical metrics.

Further details on the sampling, reporting methods, and analysis (including multiplicity, subgroups and interim analyses) will be contained within a separate statistical analysis plan (SAP). This document will be signed by all parties involved before the actual start of the analysis by the Mia software. Details of any deviations from the planned analysis or post-hoc analyses will be provided in the evaluation report.

8 SAMPLE

8.1 ELIGIBILITY CRITERIA

8.1.1 INCLUSION CRITERIA

1. Clients attending for routine breast screening purposes at NHS Grampian.
2. Clients identified as female.
3. Clients for whom a 2D FFDM standard four-view mammography examination was acquired (MLO-R, CC-R, MLO-L, CC-L).

8.1.2 EXCLUSION CRITERIA

1. Cases marked as technical recall based on the double reading final outcome.

Note: Histories of hormone replacement therapy, breast cancer and/or previous surgery for breast cancer are not exclusion criteria.

8.2 SAMPLE SIZE

The sample size is based on a non-inferiority test for the difference between the standard double reading workflow and the primary workflow with Mia (nDRT(A3)+XR(A2)) in detecting screen-detected cancers. The agreement rate between the standard double reading workflow and the primary workflow with Mia is expected to be 95.0%. A percentage of 1.9% of the confirmed positives (CPs) will be detected by the standard double reading workflow only, while 3.0% will be detected by the primary workflow with Mia. Using a one-sided alpha of 0.05 and a non-inferiority margin of 10% relative to the standard double reading workflow proportion, a sample size of 65 CPs will have a power of 91.5%. This sample size for CPs ensures that the power for the secondary cancer detection rate (CDR) endpoints for the nDRT+XR combination workflows with Mia are at least 90%.

In the time period ranging from 2016 to 2020, the service's 4-month average CDR was 8.6 per 1000 with a standard deviation of 0.9 per 1000. To ensure that the needed number of cancers will be included in the project, a tolerance interval is calculated. The lower bound of the tolerance interval that will cover 96% of the CDRs with a confidence of 95% is estimated to be 6.4 per 1000, resulting in a sample size of 10,156 screens (to obtain 65 confirmed positive cases).

8.3 EVALUATION DURATION

The project duration required to screen this number of women will be approximately 6.1 months.

9 ETHICAL AND REGULATORY CONSIDERATIONS

9.1 PPI

All information materials provided to the public have been developed and tested with clients and staff involved in breast screening to ensure that they meet clients' information needs and are easily understood. Kheiron's PPI Lead carries out ongoing work with patients and the public to produce high quality information materials for potential participants and the public, including qualitative interviews to understand their information needs and preferences. The project materials have also been reviewed by members from the Aberdeen Centre for Health Data Science (ACHDS) PPI group.

The Kheiron Patient and Public Involvement (PPI) Advisory Board will be asked to input on other aspects of the evaluation including reporting and dissemination of results.

9.2 DATA MANAGEMENT AND PROTECTION

Staff will use reasonable methods to protect the data in line with national and local regulations, and will comply with the European General Data Protection Regulation 2016/679 (GDPR).

Data will be managed by the Grampian Data Safe Haven (DaSH). Only DaSH, NHS Grampian Health staff, University of Aberdeen staff, and Kheiron members involved in analysis will have access to the data. All researchers will have up-to-date training in Good Clinical Practice and Information Governance.

The project has a DaSH DMP which provides details of how the data will be transferred, managed, stored and accessed. Researchers cannot access the internet from or transfer data files outside DaSH and printing is not possible. Outputs will be released from the safe haven following an SOP which includes a disclosure risk check. Only disclosure risk checked extracts that do not identify individuals will be released.

The Clinical Lead and all researchers who require access to the project data will sign a DaSH Investigator Declaration agreeing to the terms and responsibilities for accessing data within DaSH and is responsible for ensuring the research team is aware of their obligations.

Data will be stored for five years. After the 5 years of archiving, the DaSH team will review the status of the project data with the Data Custodian and if deletion is appropriate the data will be deleted from the University of Aberdeen and NHS servers and the backups overwritten to remove them.

The Clinical Lead and staff involved with this evaluation will not disclose or use for any purpose other than the evaluation, any data, record, or other unpublished, confidential information disclosed to those individuals for the purpose of the project. Published results will not contain any personal data that could allow identification of individual women.

Further details regarding data handling and data flows (including data required for the evaluation) are detailed in other supporting documents. A data protection impact assessment (DPIA) will be completed and approved by the Trust Data Protection Officer prior to commencement of the evaluation.

9.3 RISK ASSESSMENT

Mia has been tested in multiple clinical studies that indicate that its use in practice is likely to maintain or improve standard of screening. Mia also has a CE as a class IIa medical device for a standalone software to support diagnosis of breast cancer. Mia has been developed following a number of standards such as ISO 14971 Risk Management for Medical Devices, EN 62304 Medical Device Software Life Cycle Processes, and EN 62366 Usability Engineering to Medical Devices.

The risk to women in the evaluation is therefore expected to be minimal as all women will receive standard of care double reading according to the service's normal clinical practice. In addition, about 1 in 10 eligible women may benefit from having an additional human review of their mammogram, which is expected to result in about 38 (0.37%) additional client recalls to provide an opportunity to find more cancers and improve cancer detection rate. The additional review is conducted wherever Mia suggests to recall and standard double reading suggests to not recall (termed positive discordant). Mia is a device which has demonstrated superior performance in sensitivity and non-inferior performance in specificity compared to historical single first reading [1] and has demonstrated that its performance generalises to the service population [2]. Using Mia to triage or flag potentially positive cases for additional human review is therefore expected to help readers find more cancers in a focused review. Using Mia in this way can only result in more cancers being detected, not less, and all final screening decisions continue to be made by a human or multiple human readers.

Pre-project training can help to identify potential risks in reader behaviour changes, and screening will be reviewed in the first 1-4 months of the project or as deemed necessary for safety.

10 TRIPARTITE AGREEMENT

Intellectual property arrangements, confidentiality, publicity and publication are all covered in the tripartite agreement between the parties.

11 REFERENCES

1. Sharma, N. et al (2020). Large-scale evaluation of an AI system as an independent reader for double reading in breast cancer screening. medRxiv. doi: 10.1101/2021.02.26.21252537

2. Clarisse de Vries, Samantha Colosimo, Roger Staff et al. Artificial Intelligence in Breast Screening - Local Validation Essential, 16 May 2022, PREPRINT (Version 1) available at Research Square [https://doi.org/10.21203/rs.3.rs-1466487/v1]

APPENDIX 1: VERSION HISTORY

Protocol Version/ Amendment Number	Date	Summary of main changes
0.1	2022.10.20	N/A - Initial version
0.2	2022.11.22	Updated with further details from original protocol
0.3	2022.12.07	Edits added throughout the plan to provide further clarifications.
1.0	2023.02.01	Refinement of planned analyses; updates made to section 10 Dissemination to align with contracts

[CONFIDENTIAL]

Kheiron Medical Technologies Ltd.
PROTOCOL REFERENCE: KMT008

GEMINI: GRAMPIAN'S EVALUATION OF MIA AN INNOVATIVE NATIONAL BREAST SCREENING INITIATIVE

QUANTICS REFERENCE: 2847

STATISTICAL ANALYSIS PLAN

VERSION 2.0

DATE: 21 December 2023

CHANGE CONTROL

Date	Details	Made By:	Against version
22SEP2023	SAP version 1.0 created	Holly Trochet	–
21DEC2023	Removed mention of delivering analyses not included in TLFs in a dashboard. Corrected errors in Table 2. Corrected superiority test details to align with protocol.	Annie Ng	1.0

STATISTICAL ANALYSIS PLAN FOR KMT008

QUANTICS REFERENCE NUMBER 2847

Approved by: ________________________ Date: 12/22/2023

Annie Ng, Science Lead

Kheiron Medical Technologies Ltd

Approved by: Date: 01/09/2024

Lesley Anderson, Chair in Health Data Science

University of Aberdeen

Approved by: Date: 01/08/2024

Clarisse de Vries, Postdoctoral research fellow

University of Aberdeen

Approved by: ________________________ Date: 01/11/2024

Dr Gerald Lip

NHS Grampian

CONTENTS

CHANGE CONTROL 1
ABBREVIATIONS & DEFINITIONS 4
ABBREVIATIONS 4
SECTION 1. INTRODUCTION & TERMS 6
1.1. INTRODUCTION 6
1.2. DEFINITIONS 7
SECTION 2. STUDY OBJECTIVES AND DESIGN 10
2.1. STUDY OBJECTIVES 10
2.2. EVALUATION DESIGN 10
2.3. DETERMINATION OF SAMPLE SIZES 11
2.4. EVALUATION VISITS 11
SECTION 3. DATA 12
SECTION 4. STUDY ENDPOINTS 17
4.1. PRIMARY ENDPOINTS 19
4.2. SECONDARY ENDPOINTS 19
4.3. EXPLORATORY ENDPOINTS 19
4.4. INTERIM ANALYSES 19
SECTION 5. ANALYSIS POPULATIONS 20
SECTION 6. GENERAL POINTS FOR STATISTICAL ANALYSIS 21
6.1. GENERAL METHODS 21
6.2. DECIMAL PLACES 21
6.3. WITHDRAWALS AND MISSING DATA 21
SECTION 7. COMPLIANCE 22
SECTION 8. ANALYSIS OF STUDY ENDPOINTS 23
8.1. CONFIDENCE INTERVALS AND STATISTICAL TESTS 23
8.1.1. Wilson Confidence Interval For Proportions (Rates) 23
8.1.2. Wilson Confidence Interval For Absolute Differences 24
8.1.3. Bootstrap Confidence Intervals 24
8.1.4. Non-inferiority And Superiority Tests 24
8.1.5. Multiplicity 25
8.2. PRIMARY ENDPOINTS 26
8.3. SECONDARY ENDPOINTS 27
8.3.1. Subgroup analysis 29
8.3.2. Sensitivity of Overlapping Sets 29
8.3.3. Descriptives of Cancer Subtypes Per Workflow And Among Agreed and Discordant Cancers Between Compared Workflows 30
8.3.4. Workload Savings Compared To Double Reading Without Mia 31
8.3.5. Descriptives Of Cancer Subtypes Per Single Reader and Among Agreed and Discordant Cancers Between Single rReaders 32
8.4. EXPLORATORY ENDPOINTS 33

SECTION 9. LIST OF TABLES, LISTINGS AND FIGURES 34
REFERENCES 36

ABBREVIATIONS & DEFINITIONS

ABBREVIATIONS

AR	Arbitration rate
CAD	Computer aided detection
CC-L	Craniocaudal – left
CC-R	Craniocaudal – right
CDR	Cancer detection rate
CE	Conformité Européenne
CI	Confidence interval
CN	Confirmed negative
CP	Confirmed positive
det	Detection
DICOM	Digital Imaging and Communications in Medicine
DPIA	Data Protection Impact Assessment
DR	Double reading (workflow)
FFDM	Full Field digital Mammography
GCP	Good Clinical Practice
GDPR	General Data Protection Regulation
INV	Invasive cancer
Ltd	Limited
MLO-L	Mediolateral oblique – left
MLO-R	Mediolateral oblique – right
NBSS	National Breast Screening System
nDRT	Double reader triage (workflow)
NFR	Negative flag rate
nIR	Independent reader (workflow)
NP	Non-positive case

OP	Operating point
PACS	Picture Archiving and Communication System
PPI	Patient and Public Involvement
PPV	Positive predictive value
pred	Prediction
ROI	Region(s) of interest
R1	Human reader 1
R2	Human reader 2
RR	Recall rate
SA	Stand alone
SAP	Statistical Analysis Plan
SEN	Sensitivity
SPEC	Specificity
SR	Silent reader
SR/XR	Silent reader/extra reader combination
UUID	Universally unique identifier
XR	Extra reader (workflow)

Section 1. Introduction & Terms

1.1. Introduction

This document describes the statistical analysis and reporting plan for data from GEMINI: Grampian's Evaluation of Mia an Innovative National breast screening Initiative [1]. This project will evaluate the safety and efficacy that Mia, an Artificial Intelligence (AI) software medical device, can bring to the breast cancer screening workflow through opportunities of clinical and operational benefit. This project will evaluate the performance of Mia integrated into double reading for breast cancer screening compared to standard double reading without Mia.

The evaluation will evaluate the use of Mia for two main purposes:

1. 'Triage Negatives' to provide workload savings
2. 'Triage Positives' to provide opportunities for increased cancer detection.

As described in the ICH E9 guideline [2], the purpose of this document is to provide a more technical and detailed elaboration of the principal features of the analysis described in the study protocol, and to include detailed procedures for executing the statistical analysis of the primary and secondary variables and other data.

Details of the procedures that will be followed by Quantics when undertaking data receipt, analysis and reporting are described in Quantics SOPs [3] [4].

Any amendments to the SAP will be made prior to database lock.

Any additional analyses not described in the final SAP or deviations from the final SAP will be documented in the clinical study report.

1.2. DEFINITIONS

**Confirmed negative cases (CN)**	Cases where a negative follow-up result is available which includes mammography reading beyond a time period based on the screening interval (expected c.34 months) without sign of malignancy between.
**Confirmed positive cases (CP)**	Cases where a malignancy is confirmed by a decisive biopsy, cytology or histology of the surgical specimen downstream within a follow-up period (expected approximately 2-3 months)
**Core project time period**	Time period between the first and last case recruited into the evaluation.
**Incident**	Incident cases i.e. cases where participants have a previous (prevalent) screen.
**Invasive cancers (inv)**	Cancers which are considered invasive.
**Mia™**	CE marked medical device indicated to aid readers in the interpretation of breast imaging examinations for the early detection and diagnosis of breast cancer.
**Negative flagged**	A case deemed not to require double reading with R2 when Mia and R1 agree no recall, in the context of the double reader triage (nDRT) workflow.
**Negative discordant cases**	Cases where Mia (SA) suggests no recall and the double reading (DR) opinion is to recall.
**Non-positive cases (NP)**	Cases where a malignancy is not confirmed within a 'positive evidence collection period'.
**Operating point (OP)**	A pre-determined decision threshold for the Mia software that specifies a tradeoff between sensitivity and specificity.
**Positive discordant cases**	Cases where Mia (SA) suggests to recall and the double reading (DR) opinion is to not recall.
**Positive evidence collection period**	Period after the last case recruited into the evaluation (i.e. the core project time period), where evidence of malignancy and cancer subtype information will be collected for cases (circa 2-3 months).
**Prevalent**	Prevalent cases i.e. cases where participants present for their first screen without history of a previous screening episode.
**Total number of cases**	The total number of sampled cases relevant for an endpoint (varies by endpoint).

Workflows

**Combination workflow**	A combination of nDRT or nIR workflows with the XR workflow, at given operating points.
**Consensus arbitration**	Arbitration of cases where two or more readers agree to recall in order to make a final double reading decision according to local standard operating practice.
**Discordance arbitration**	Arbitration of cases where two or more readers disagree in order to make a final double reading decision according to local standard operating practice.
**Double reader triage (nDRT)**	A double reading workflow utilising Mia as an independent second reader in cases where Mia and the first reader (R1) agree to not recall. Otherwise, a second human reader (R2) serves as the second reader. Double reading is carried out using R1, either Mia or R2, and arbitration when required. This workflow is analysed in this evaluation using Mia operating points A2, A3, and A4 at the end of the core project time period.
**Double reading (DR)**	A workflow where two readers (R1, R2) read every case. When R1 and R2 agree to not recall, the case is not recalled. When R1 and R2 disagree, discordance arbitration is conducted to make the final double reading decision. When R1 and R2 agree to recall, arbitration may also be conducted to make the final double reading decision, depending on the site's arbitration practice in what is called 'consensus arbitration'.
**Double reading with Mia**	A double reading workflow that incorporates Mia as an independent reader. Specifically, in this evaluation, this includes double reader triage (nDRT), independent reader (nIR), extra reader (XR), nDRT+XR, and nIR+XR.
**Extra reader (XR)**	A double reading workflow where Mia's additional read serves as a third independent read. 'Positive discordant' cases are then subsequently reviewed by a human reader or group of human readers depending on local practice in an 'extra arbitration' step where the case is additionally reviewed. During 'extra arbitration', readers will have access to the AI output, prior arbitration decision and prior mammograms/clinical notes, and will review the case with the aim of detecting additional cancers. This workflow will primarily use Mia operating point A2 in the evaluation but results will also be generated for operating point A1 via simulation.
**Independent reader (configuration)**	Configuration where Mia serves as an independent reader. Potential workflows in this evaluation include extra reader (XR), double reader triage (nDRT), independent reader (nIR), and combination workflows.
**Independent reader (nIR) (workflow)**	A double reading workflow where Mia is used as the second reader when R1 and Mia agree. If they disagree, Mia's opinion is disregarded and the Reader 2 (R2) decision is used, thus the workflow defaults to standard double reading. This workflow is sometimes referred to as 'non-interactive independent reader' (nIR). This workflow is simulated in this evaluation using Mia operating points A1 and A2.

Standalone (SA) Device's case-wise malignancy assessment independent of any workflow or radiologist opinion. In this evaluation, Mia standalone performance will be assessed at operating points A1, A2, A3, and A4.

Metrics

Arbitration rate (AR) The number of cases arbitrated divided by the total number of cases.

Cancer detection rate (CDR) The number of positive cases recalled divided by the total number of cases.

Negative flag rate (NFR) Metric associated with the nDRT workflow; the number of cases 'negative flagged' (i.e. not deemed requiring double reading with R2 when Mia and R1 agree no recall) divided by the total number of cases.

Negative predictive value (NPV) The number of confirmed negative (CN) cases not recalled divided by the total number of cases not recalled.

Positive discordance rate The number of positive discordant cases divided by the total number of cases.

Positive predictive value (PPV) The number of positive cases recalled divided by the total number of cases recalled.

Recall rate (RR) The number of cases recalled divided by the total number of cases.

Recall rate on positive discordant cases (RRpd) The number of positive discordant cases recalled during 'extra arbitration' in the XR workflow divided by the total number of positive discordant cases.

Sensitivity (SEN) The number of positive cases recalled divided by the total number of positive cases.

Specificity (SPEC) The number of confirmed negative (CN) cases not recalled divided by the total number of confirmed negative cases.

Specificity on non-positives (SPECnp) The number of non-positive cases not recalled divided by the total number of non-positive cases.

Section 2. Evaluation Objectives and Design

2.1. Study Objectives

The primary aim of this evaluation is to assess the safety and efficacy that Kheiron's software, Mia, can bring to the breast cancer screening workflow through opportunities of clinical and operational benefit. The objectives are to

- Deploy Mia in the extra reader (XR) workflow within the breast screening service.
- Silently run Mia in double reading workflows to retrospectively analyse their potential impact and contribution to the breast screening service.
- Collect data from Mia, the Scottish Breast Screening System (SBSS), and screening mammograms to analyse key clinical outcomes in breast screening including possible workload savings in breast screening.

2.2. Evaluation Design

This project will evaluate the safety and efficacy of the use of Mia, an Artificial Intelligence (AI) software medical device, in double reading for breast cancer screening compared to standard double reading without Mia in a paired design.

The evaluation will evaluate the use of Mia for two main purposes; to:

1. 'Triage Negatives' to provide workload savings, and to
2. 'Triage Positives' to provide opportunities for increased cancer detection.

The primary double reading workflow with Mia to be evaluated is a combination of two workflows, 'double reader triage' and 'extra reader', which serve to 'triage negatives' and 'triage positives', respectively. **The primary outcome will evaluate the combined workflow of 'Mia double reader triage' at operating point A3 and 'Mia extra reader' at operating point A2 ('primary workflow').**

The first part of the workflow, to **'triage negatives'**, will have **no impact** on the service as the workflow will be exactly simulated during retrospective analysis. The service can conduct standard double reading according to their routine clinical practice during the evaluation.

The second part of the workflow, to **'triage positives'**, occurs after the service's full double reading workflow is performed with their standard arbitration practice. In this part of the workflow, Mia is used as an **extra reader**, flagging cases where Mia suggests to recall which double reading did not suggest to recall (termed 'positive discordant' cases). These flagged positive discordant cases will undergo additional review by experienced readers within the service with access to the AI output, prior arbitration decision and prior mammograms/clinical notes. If the readers determine that the AI has detected a case that

requires further evaluation, the client will be recalled and offered a follow-up appointment, providing an opportunity to find more cancers.

Variants of the primary workflow will also be analysed at various operating point combinations. Further, each of the workflow components will also be analysed independently at relevant operating points to understand their impact and contribution to the combined workflows.

2.3. DETERMINATION OF SAMPLE SIZES

The sample size is based on a non-inferiority test for the difference between the standard double reading workflow and the primary workflow with Mia (nDRT(A3)+XR(A2)) in detecting screen-detected cancers. The agreement rate between the standard double reading workflow and the primary workflow with Mia is expected to be 95.0%. A percentage of 1.9% of the confirmed positives (CPs) will be detected by the standard double reading workflow only, while 3.0% will be detected by the primary workflow with Mia. Using a one-sided alpha of 0.05 (this equals the lower bound of the two-sided 90% CI) and a non-inferiority margin of 10% relative to the standard double reading workflow proportion, a sample size of 65 CPs will have a power of 91.5%. This sample size for CPs ensures that the power for the secondary endpoints for the nDRT+XR combination workflows with Mia is at least 90%.

In the time period ranging from 2016 to 2020, the service's 4-month average CDR was 8.6 per 1000 with a standard deviation of 0.9 per 1000. To ensure that the needed number of cancers will be included in the project, a tolerance interval is calculated. The lower bound of the tolerance interval that will cover 96% of the CDRs with a confidence of 95% is estimated to be 6.4 per 1000, resulting in a sample size of 10,156 screens (to obtain 65 confirmed positive cases).

- If 1.9% of CPs is detected by standard double reading only, this equals a CDR of 0.12/1000.
- If 3.0% of CPs is detected by the primary Mia workflow only, this equals a CDR of 0.2/1000.

Non-inferiority hypothesis testing will be performed using the CDR values.

2.4. EVALUATION VISITS

Site visits will be made as deemed necessary during the evaluation.

Section 3. DATA

All cases are expected to be organised as follows.

- Rows: each row in the dataset is expected to belong to one case
- Columns: there will be a column for each of the variables described in Table 1 below.

Table 1: List of variables that will make up the raw data.

**Variable description**	**Variable name**	**Description of potential values**
Site ID	site	Unique identifier of the hospital/medical center where the screen took place.
Subject ID	patient_uid	Unique subject identifier.
Case ID	case_uid	De-identified (unique) ID for the case (subject at a given timepoint) to which the images belong.
Vendor ID	Vendor	Unique identifier of the hardware vendor.
Subject age at mammogram	age_at_case	The age of the participant to whom the images belong at the time of image acquisition
Subject sex	sex	The sex of the participant to whom the images belong at the time of image acquisition
Breast density of case by algorithm 1, 2, 3 etc. as applicable	breast_density_algorithm1, breast_density_algorithm2, breast_density_algorithm3 etc. as applicable	Breast density decision of the case by algorithm 1, 2, 3, etc. as applicable
Date of mammogram	date	Date of screen in format 01JAN2022.
Confirmed positive	cp	0/1 flag; 1 if the patient is confirmed positive, 0 if not based on complete positive evidence collection. NA if patient lost to follow-up during the positive evidence collection period during which the decisive biopsy, cytological or histological study should have been performed.
Non-positive	np	0/1 flag; 1 if there is no malignancy confirmed for the patient based on complete positive evidence collection. 0 if not based on complete positive evidence collection. NA if patient lost to follow-up during the positive evidence collection period during which the decisive biopsy, cytological or histological study should have been performed.

Variable description	Variable name	Description of potential values
Incident or prevalent case	inc_prev	"prev"/"inc" flag; "prev" if this is an prevalent case, "inc" if it is incident.
Read time for screening	read_time_screen	Numerical and time bracket value
Read time for double reading arbitration	read_time_arbitration	Numerical and time bracket value
Read time for XR arbitration	read_time_xr	Numerical and time bracket value
Mia decision for the standalone workflow at operating point A1	sa_decision_mia_a1	0/1 flag; 1 if the decision was to recall, 0 if the decision was not to recall.
Mia decision for the standalone workflow at operating point A2	sa_decision_mia_a2	0/1 flag; 1 if the decision was to recall, 0 if the decision was not to recall.
Mia decision for the standalone workflow at operating point A3	sa_decision_mia_a3	0/1 flag; 1 if the decision was to recall, 0 if the decision was not to recall.
Mia decision for the standalone workflow at operating point A4	sa_decision_mia_a4	0/1 flag; 1 if the decision was to recall, 0 if the decision was not to recall.
Arbitrated in the double reader workflow	arb_dr	0/1 flag; 1 if the decision was sent to an arbitrator in the double reader workflow, 0 if not.
Arbitrator decision in the double reading workflow	arb_dr_decision	0/1/NA flag; 1 if the arbitrator's decision in the double reader workflow was to recall, 0 if the decision was not to recall, NA if the arbitrator was not used.
Decision for the double reader (DR) workflow	dr_decision	0/1 flag; 1 if the decision was to recall, 0 if the decision was not to recall.
Decision of the first reader	r1_decision	0/1 flag; 1 if the decision was to recall, 0 if the decision was not to recall.
Decision of the second reader	r2_decision	0/1 flag; 1 if the decision was to recall, 0 if the decision was not to recall.
XR Arbitrator decision on positive discordant cases at Mia operating point A1	arb_xr_decision_a1	0/1 flag; 1 if the XR arbitrator decision was to recall, 0 if the decision was not to recall. NA if the case was not arbitrated as part of XR.
XR Arbitrator decision on positive discordant cases at Mia operating point A2	arb_xr_decision_a2	0/1 flag; 1 if the XR arbitrator decision was to recall, 0 if the decision was not to recall. NA if the case was not arbitrated as part of XR.
Final decision in the extra reader (XR) workflow at Mia operating point A1	xr_decision_mia_a1	0/1 flag; 1 if the decision of the xr workflow was to recall, 0 if the decision was not to recall.

Variable description	Variable name	Description of potential values
Final decision in the extra reader (XR) workflow at Mia operating point A2	xr_decision_mia_a2	0/1 flag; 1 if the decision of the xr workflow was to recall, 0 if the decision was not to recall.
Arbitrated in the XR workflow (i.e. positive discordant cases) at Mia operating point A1	arb_xr_mia_a1	0/1 flag; 1 if the decision was sent to an XR arbitrator in the XR workflow at Mia OP A1, 0 if not.
Arbitrated in the XR workflow (i.e. positive discordant cases) at Mia operating point A2	arb_xr_mia_a2	0/1 flag; 1 if the decision was sent to an XR arbitrator in the XR workflow at Mia OP A2, 0 if not.
Decision in the double reader triage (nDRT) workflow at Mia operating point A2	ndrt_decision_mia_a2	0/1 flag; 1 if the decision was to recall, 0 if the decision was not to recall.
Decision in the double reader triage (nDRT) workflow at Mia operating point A3	ndrt_decision_mia_a3	0/1 flag; 1 if the decision was to recall, 0 if the decision was not to recall.
Decision in the double reader triage (nDRT) workflow at Mia operating point A4	ndrt_decision_mia_a4	0/1 flag; 1 if the decision was to recall, 0 if the decision was not to recall.
Negative flagged (nDRT workflow) at Mia operating point A2	negative_flagged_a2	0/1 flag; 1 if the case is negative flagged, 0 if it is not.
Negative flagged (nDRT workflow) at Mia operating point A3	negative_flagged_a3	0/1 flag; 1 if the case is negative flagged, 0 if it is not.
Negative flagged (nDRT workflow) at Mia operating point A4	negative_flagged_a4	0/1 flag; 1 if the case is negative flagged, 0 if it is not.
Decision sent to an arbitrator in the double reader triage (nDRT) workflow at Mia operating point A2	arb_ndrt_mia_a2	0/1 flag; 1 if the decision was sent to an arbitrator in the nDRT workflow at Mia OP A2, 0 if not.
Decision sent to an arbitrator in the double reader triage (nDRT) workflow at Mia operating point A3	arb_ndrt_mia_a3	0/1 flag; 1 if the decision was sent to an arbitrator in the nDRT workflow at Mia OP A3, 0 if not.
Decision sent to an arbitrator in the double reader triage (nDRT) workflow at Mia operating point A4	arb_ndrt_mia_a4	0/1 flag; 1 if the decision was sent to an arbitrator in the nDRT workflow at Mia OP A4, 0 if not.

Variable description	Variable name	Description of potential values
Decision for the independent reader (nIR) workflow at Mia operating point A1	nir_decision_mia_a1	0/1 flag; 1 if the decision was to recall, 0 if the decision was not to recall.
Decision for the simulated independent reader (nIR) workflow at Mia operating point A2	nir_decision_mia_a2	0/1 flag; 1 if the decision was to recall, 0 if the decision was not to recall.
Decision sent to an arbitrator in the independent reader (nIR) workflow at Mia operating point A1	arb_nir_mia_a1	0/1 flag; 1 if the decision was sent to an arbitrator in the nIR workflow at Mia OP A1, 0 if not.
Decision sent to an arbitrator in the independent reader (nIR) workflow at Mia operating point A2	arb_nir_mia_a2	0/1 flag; 1 if the decision was sent to an arbitrator in the nIR workflow at Mia OP A2, 0 if not.
Decision for the nDRT+XR workflow at Mia operation points A2 and A1, respectively	ndrt_xr_decision_mia_a2_a1	0/1 flag; 1 if the decision was to recall, 0 if the decision was not to recall.
Decision for the nDRT+XR workflow at Mia operation points A2 and A2, respectively	ndrt_xr_decision_mia_a2_a2	0/1 flag; 1 if the decision was to recall, 0 if the decision was not to recall.
Decision for the nDRT+XR workflow at Mia operation points A3 and A1, respectively	ndrt_xr_decision_mia_a3_a1	0/1 flag; 1 if the decision was to recall, 0 if the decision was not to recall.
Decision for the nDRT+XR workflow at Mia operation points A3 and A2, respectively	ndrt_xr_decision_mia_a3_a2	0/1 flag; 1 if the decision was to recall, 0 if the decision was not to recall.
Decision for the nDRT+XR workflow at Mia operation points A4 and A1, respectively	ndrt_xr_decision_mia_a4_a1	0/1 flag; 1 if the decision was to recall, 0 if the decision was not to recall.
Decision for the nDRT+XR workflow at Mia operation points A4 and A2, respectively	ndrt_xr_decision_mia_a4_a2	0/1 flag; 1 if the decision was to recall, 0 if the decision was not to recall.
Decision for the nIR+XR workflow at Mia operation points A1 and A1, respectively	nir_xr_decision_mia_a1_a1	0/1 flag; 1 if the decision was to recall, 0 if the decision was not to recall.

Variable description	Variable name	Description of potential values
Decision for the nIR+XR workflow at Mia operation points A1 and A2, respectively	nir_xr_decision_mia_a1_a2	0/1 flag; 1 if the decision was to recall, 0 if the decision was not to recall.
Decision for the nIR+XR workflow at Mia operation points A2 and A1, respectively	nir_xr_decision_mia_a2_a1	0/1 flag; 1 if the decision was to recall, 0 if the decision was not to recall.
Decision for the nIR+XR workflow at Mia operation points A2 and A2, respectively	nir_xr_decision_mia_a2_a2	0/1 flag; 1 if the decision was to recall, 0 if the decision was not to recall.
Invasivity based on histologic type (tissue diagnosis) from biopsy or surgeryCancer invasiveness flag	cancer_invasive	0/1 flag; 1 if the cancer is invasive, 0 if it is not. NA if there is no cancer.
Tumour type for invasive cancers	invasive_tumour_type	List including: Ductal/NST, Tubular/Cribriform, Medullary-Like, Mucinous, Lobular, Micropapillary, Other, Unclassifiable
Invasive tumour size	invasive_tumor_size	Numerical value; invasive tumour size (in mm) based on imaging.
Histological grade for invasive cancers	invasive_histological_grade	grade low, intermediate, high; not_assessable; NA if information is missing.
Lymph node status for invasive cancers	lymph_node_status	TBC
Tumour type for non-invasive cancers	non_invasive_tumour_type	"Ductal"; "Lobular"; NA if information is missing.
Histological grade for non-invasive cancers	non_invasive_histological_grade	grade low, intermediate, high; not_assessable; NA if information is missing.
ER receptor status	er_status	“positive” if positive ER status, “negative” if not. NA if information is missing.
PgR receptor status	pgr_status	“positive” if positive PgR status, “negative” if not. NA if information is missing.
HER-2 IHC score	her2_ihc_score	TBC
HER-2 FISH	her2_fish	TBC
Final HER-2 status	final_her2_status	TBC

Section 4. Study Endpoints

This study makes use of multiple workflows, which are combinations of Mia and human readers used to determine whether or not to recall someone. These workflows are detailed in Section 1.2. Additionally, Mia can be set to different operating points, which specify the decision thresholds for the software. The results of each workflow at the relevant operating points will be recorded in the data. Tables 2 and 3 below summarise the primary (in Table 3 only), secondary, and exploratory endpoints. The primary endpoint is denoted with **P***, secondary with S, exploratory with E. Additional details and endpoints are listed in the sections below. The secondary endpoints highlighted in blue in Table 2 will additionally be assessed in interim analyses described in section 3.4.

Table 2: Endpoint values to be recorded for each workflow and each operating point (OP). Endpoints highlighted in blue will be additionally covered in interim analyses.

Measurement Endpoints for Overall																								
Configuration / Workflow	*Mia SA*				*R1*	*R2*	*Mia nDRT*			*Mia nIR*		*Mia XR*		*Mia nDRT+XR*						*Mia nIR+XR*				*DR*
Mia OP	*A1*	*A2*	*A3*	*A4*	*N/A*	*N/A*	*A2*	*A3*	*A4*	*A1*	*A2*	*A1*	*A2*	*A2/ A2*	*A2/ A1*	*A3/ A2*	*A3/ A1*	*A4/ A2*	*A4/ A1*	*A1/ A2*	*A1/ A1*	*A2/ A2*	*A2/ A1*	*N/A*
NFR							S	S	S															
Reduction in portion of cases read by R2 (compared to DR)							S	S	S	S	S			S	S	S	S	S	S	S	S	S	S	
Reduction in portion of cases read by Arb (compared to DR)							S	S	S	S	S			S	S	S	S	S	S	S	S	S	S	
Workload savings based on measured reading times (as available) and if Arb read time is 1x, 2x, 3x, 4x first reads time							S	S	S	S	S			S	S	S	S	S	S	S	S	S	S	
AR							S	S	S	S	S			S	S	S	S	S	S	S	S	S	S	
Positive Discordant Rate												S	S	S	S	S	S	S	S	S	S	S	S	
Negative Discordant Rate												S	S	S	S	S	S	S	S	S	S	S	S	
RR among positive discordant												S	S	S	S	S	S	S	S	S	S	S	S	
RR	S	S	S	S	S	S	S	S	S	S	S	S	S	S	S	S	S	S	S	S	S	S	S	S
CDR	S	S	S	S	S	S	S	S	S	S	S	S	S	S	S	S	S	S	S	S	S	S	S	S
SEN	S	S	S	S	S	S	S	S	S	S	S	S	S	S	S	S	S	S	S	S	S	S	S	S
PPV	S	S	S	S	S	S	S	S	S	S	S	S	S	S	S	S	S	S	S	S	S	S	S	S
SPECnp	S	S	S	S	S	S	S	S	S	S	S	S	S	S	S	S	S	S	S	S	S	S	S	S
SEN (Intersection of Mia SA & R1, Mia only, R1 only)	S	S	S	S	S																			
SEN (Intersection of Mia + DR workflow, Mia workflow only, DR only)							S	S	S	S	S	S	S	S	S	S	S	S	S	S	S	S	S	S
Descriptives of cancer subtypes	S	S	S	S	S	S	S	S	S	S	S	S	S	S	S	S	S	S	S	S	S	S	S	S

Descriptives of cancer subtypes among positive discordant												*S*	*S*	*S*	*S*	*S*	*S*	*S*	*S*	*S*	*S*	*S*	*S*	*S*
Subgroup analyses	*E*	*E*	*E*	*E*	*E*	*E*	*S*	*S*	*S*	*S*	*S*	*S*	*S*	*S*	*S*	*S*	*S*	*S*	*S*	*S*	*S*	*S*	*S*	*S*

Mia SA = Mia Standalone; R1 = reader 1; R2 = reader 2; nDRT = double reader triage; XR = extra reader; DR = double reader; OP = operating point; NFR = negative flag rate; Arb = arbitrator(s); AR = arbitration rate; RR = recall rate; CDR = cancer detection rate; SEN = sensitivity; PPV = positive predictive value; SPECnp = specificity on non-positives; S = secondary; E = exploratory.

Table 3: Non-inferiority and superiority tests and absolute and relative differences to DR, to be recorded for each endpoint and each operating point (OP).

Non-inferiority and Superiority Test Endpoints (including absolute and relative differences) for OVERALL										
**Configuration / Workflow**	**Mia nDRT+XR compared to DR**						**Mia nIR+XR compared to DR**			
**Mia OP**	**A2/ A2**	**A2/ A1**	**A3/ A2**	**A3/ A1**	**A4/ A2**	**A4/ A1**	**A1/ A2**	**A1/ A1**	**A2/ A2**	**A2/ A1**
**NFR**										
**Reduction in portion of cases read by R2 (compared to DR)**										
**Reduction in portion of cases read by Arb (compared to DR)**										
**Workload savings based on measured reading times (as available) and if Arb read time is 1x, 2x, 3x, 4x first reads time**										
**AR**	S	S	S	S	S	S	S	S	S	S
**Positive Discordant Rate**										
**Negative Discordant Rate**										
**RR among positive discordant**										
**RR**	S	S	S	S	S	S	S	S	S	S
**CDR**	S	S	**P***	S	S	S	S	S	S	S
**SEN**	S	S	S	S	S	S	S	S	S	S
**PPV**	S	S	S	S	S	S	S	S	S	S
**SPECnp**	S	S	S	S	S	S	S	S	S	S
**% cancers detected (Mia SA & R1, Mia only, R1 only)**										
**% cancers detected (Mia + DR workflow, Mia workflow only, DR only)**										
**Descriptives of cancer subtypes**										
**Descriptives of cancer subtypes among positive discordant**										
**Subgroup analyses**										

R1 = reader 1; R2 = reader 2; nDRT = double reader triage; XR = extra reader; DR = double reader; OP = operating point; NFR = negative flag rate; Arb = arbitrator(s); AR = arbitration rate; RR = recall rate; CDR = cancer detection rate; SEN = sensitivity; PPV = positive predictive value; SPECnp = specificity on non-positives; S = secondary; P* = primary.

4.1. Primary Endpoints

At the end of the positive evidence collection time period, a non-inferiority test (and estimates of the associated absolute and relative differences) will be performed for cancer detection rate (CDR) between standard double reading (without Mia) and a combination of 'double reader triage' (nDRT) using Mia at OP A3 and 'extra reader' (XR) using Mia at OP A2 (nDRT(A3)+XR(A2)).

4.2. Secondary Endpoints

The secondary endpoints are denoted in Tables 2 and 3 with the letter S. For the secondary endpoints in Tables 2 and 3, the point estimate and 90% confidence interval (calculated as described in Section 8.1) will be reported. Further details of other secondary endpoints can be found in Section 8.3.

4.3. Exploratory Endpoints

Exploratory endpoints are subgroup analyses for endpoints reported for single readers R1, R2, and Mia Standalone (Table 2). They are detailed in Section 8.4.

4.4. Interim Analyses

The secondary endpoints highlighted in blue in table 2 will additionally be assessed in interim analyses before the positive evidence collection period is complete. The interim analyses in Table 2 will be done for a portion of the evaluation time period where positive follow-up information is sufficiently complete based on the data collected for the interim analyses for all selected metrics. Additionally, metrics not requiring positive follow-up information will be based on data collected for the interim analyses even if positive follow-up information is not complete. Also, the number of additional cancers found in the XR workflow on top of DR will be assessed.

In the circumstance that unexpected deviations from the study workflow occur e.g. by technical error, interim analyses may be conducted to assess their impact on the evaluation.

All planned interim analyses are measurements, not statistical tests. Therefore no correction for multiplicity is required.

Section 5. Analysis Populations

All data sent will be pooled and analysed. There will be no separate analysis populations as this is single site evaluation.

Section 6. General Points for Statistical Analysis

6.1. General Methods

All endpoints will be expressed as ratios of frequencies, and, unless otherwise stated, the point estimates and their two-sided 90% and 95% confidence intervals will be calculated and reported.

All statistical analyses will be performed using programming languages (for example, R [5] and Python [6]).

Categorical variables will be summarized as frequencies and ratios.

For continuous variables, the mean, median, first and third quartiles, and maximum and minimum will be reported.

The number of non-missing and missing datapoints will be reported for all variables.

6.2. Decimal Places

Percentages will be displayed to one decimal place. They will not be presented when the count is zero, and 100% will be presented as an integer.

Rates will be displayed with 3 decimal places for point estimates and 4 decimal places for confidence intervals.

Percentage differences, differences of ratios, and quotients of ratios will be displayed with 3 decimal places for point estimates and 4 decimal places for confidence intervals.

6.3. Withdrawals and Missing Data

As long as inclusion and exclusion criteria are met, all data will be analysed. Cases where Mia does not produce any output will be excluded from the analysis. In a real life scenario, a participant of screening with a non-valid output from Mia would default to standard double reading without Mia.

Section 7. COMPLIANCE

Analyses will be performed according to the SAP. Where there is a deviation, the sponsor will be alerted by e-mail and asked to advise on how to proceed. If analyses deviate from the SAP, this will be noted in the analysis report.

Section 8. Analysis of Study Endpoints

This study uses Mia and human readers both independently and in a number of different combinations, or "workflows". The metrics defined below form the basis of many of the study endpoints. For a given set X, we define $|X|$ as the number of elements (size) of X. For two sets, A and B, $A \cup B$ is their union; and $A \cap B$ is their intersection. Then we provide the general equations for each of the metrics below:

$$Arbitration\ rate\ (AR) = \frac{|arbitrated|}{|analysed|} \tag{1}$$

$$Recall\ rate\ (RR) = \frac{|recalled\ by\ workflow|}{|analysed|} \tag{2}$$

$$Positive\ discordance\ rate = \frac{|positive\ discordant|}{|analysed|} \tag{3}$$

$$Recall\ rate\ on\ positive\ discordant\ cases\ (RRpd) = \frac{|recalled\ by\ workflow \cap positive\ discordant|}{|positive\ discordant|} \tag{4}$$

$$Negative\ flag\ rate\ (NFR) = \frac{|negative\ flagged|}{|analysed|} \tag{5}$$

$$Cancer\ detection\ rate\ (CDR) = \frac{|confirmed\ positive \cap recalled\ by\ workflow|}{|analysed|} \tag{6}$$

$$Sensitivity\ (SEN) = \frac{|confirmed\ positive \cap recalled\ by\ workflow|}{|confirmed\ positive|} \tag{7}$$

$$Specificity\ on\ non-positives\ (SPECnp) = \frac{|non-positive \cap no\ recall\ by\ workflow|}{|non-positive|} \tag{8}$$

$$Positive\ predictive\ value\ (PPV) = \frac{|confirmed\ positive \cap recalled\ by\ workflow|}{|recall|} \tag{9}$$

$$Negative\ predictive\ value\ (NPV) = \frac{|confirmed\ negative \cap no\ recall\ by\ workflow|}{|no\ recall|} \tag{10}$$

8.1. CONFIDENCE INTERVALS AND STATISTICAL TESTS

8.1.1. WILSON CONFIDENCE INTERVAL FOR PROPORTIONS (RATES)

All calculated proportions (rates) should be paired with 90% and 95% Wilson confidence intervals [7]. Define

- $\hat{p}$ is the observed proportion and $\hat{q} = 1 - \hat{p}$.

- $\kappa = \Phi^{-1}\left(1 - \frac{\alpha}{2}\right)$ is the appropriate value from the Z distribution, while α, the type I error rate, is 0.1 in this case.
- n is the proportion denominator ('number of trials').

Then for a proportion, p, its Wilson confidence interval, CI_W, is given as follows.

$$CI_W = \frac{n\hat{p} + \frac{\kappa^2}{2}}{n + \kappa^2} \pm \frac{\kappa\sqrt{n}}{n + \kappa^2}\sqrt{\hat{p}\hat{q} + \frac{\kappa^2}{4n}}$$

This interval is the default interval calculated when using the `binom.confint()` function in the `binom` R package, which is how intervals will be calculated for the R branch of the analysis.

8.1.2. Wilson Confidence Interval For Absolute Differences

CIs for differences in proportions will be calculated using an adaptation of the Wilson method due to Newcombe [8].

For two proportions p_A and p_B, if we denote their lower and upper Wilson CI limits by L_A, U_A and L_B, U_B respectively, then the confidence interval for the difference $p_A - p_B$ has lower and upper limits L_{AB} and U_{AB} which are given by:

$$L_{AB} = \left(\hat{p}_A - \hat{p}_B\right) - \sqrt{\left(\hat{p}_A - L_A\right)^2 + \left(U_B - \hat{p}_B\right)^2}$$

$$U_{AB} = \left(\hat{p}_A - \hat{p}_B\right) + \sqrt{\left(U_A - \hat{p}_A\right)^2 + \left(\hat{p}_B - L_B\right)^2}$$

8.1.3. Bootstrap Confidence Intervals

A two-sided 90% CI for a relative difference will be calculated using bootstrapping. In addition, a one-sided 90% CI will be calculated.

The number of bootstrap iterations will need to be sufficient to ensure replicability of results (using different random seed values) to a precision of four decimal places. If this is not achievable within a reasonable number of iterations, as determined by the responsible statistician, then results will be printed to fewer decimal places.

8.1.4. Non-inferiority and Superiority Tests

For a given metric, *M*, and workflows *A* and *B*, we compare the performance of *M* at workflow *A* (M_A) to *M* at workflow *B* (M_B) using the relative difference, which is

$$Relative\ difference = \frac{M_A}{M_B}.$$

In general, M_A will be the Mia outcome at a given OP for that metric and M_B will be the historical double reader outcome. The two-sided 90% CIs will be reported, while the non-inferiority tests will be performed on the relevant bound of the CI (i.e. one-sided test with an alpha of 0.05). All tests will be performed against a relative margin of 0.1; that is the smaller ratio must always be at least 90% of the larger one, regardless of which of M_A or M_B is the smaller ratio.

Wherever the non-inferiority test passes, a superiority test of the relative difference, using the one-sided 90% CIs will be calculated and reported. Superiority will be declared if the confidence interval does not include 1.

For RR, a lower value is better. For any other rate, a higher value is better. Non-Inferiority and superiority will be assessed as in Table 4.

Non-inferiority and superiority will be assessed using the relative difference. The absolute difference with its 90% CI will also be reported (calculated per Section 8.1.2), but will not have any hypothesis test associated with it.

Table 4: Summary of non-inferiority and superiority calculations.

**Hypothesis**	**Comparison**	**Reported CI for ratios**	**Metric type**	**Difference**	**Test**	**Alpha**
Non-inferiority	Double reading	two-sided 90%	Higher is better	Relative	LCL > 0.90	0.05
Non-inferiority	Double reading	two-sided 90%	Lower is better	Relative	UCL < (1/0.90)	0.05
Superiority	Double reading	one-sided 90%	Higher is better	Relative	LCL > 1	0.1
Superiority	Double reading	one-sided 90%	Lower is better	Relative	UCL < 1	0.1
Abbreviations: LCL (Lower Confidence Limit); UCL (Upper Confidence Limit)						

8.1.5. MULTIPLICITY

A gating strategy is defined per workflow per operating point combo for the non-inferiority and superiority testing. The order of the tests is defined a priori. There will be no corrections applied for alpha. If a test is not passed, the next test will be exploratory instead of confirmatory.

Table 5: Gating strategy per workflow per operating point.

	**nDRT A3**		**nDRT A4**		**nDRT A2**		**NIR A2**		**NIR A1**	
**Gating order**	**XR A2**	**XR A1**	**XR A2**	**XR A1**	**XR A2**	**XR A1**	**XR A2**	**XR A1**	**XR A2**	**XR A1**

1	CDR (P)	CDR (P)	RR	RR	RR	RR	RR	RR	RR	RR
2	RR	RR	SPECnp	SPECnp	SPECnp	SPECnp	SPECnp	SPECnp	SPECnp	SPECnp
3	SEN	SEN	SEN	SEN	PPV	PPV	SEN	SEN	SEN	SEN
4	SPECnp	SPECnp	CDR	CDR	SEN	SEN	CDR	CDR	CDR	CDR
5	PPV	PPV	PPV	PPV	CDR	CDR	PPV	PPV	PPV	PPV

8.2. PRIMARY ENDPOINTS

We will calculate the cancer detection rate (CDR) using Equation 6. For the standard double reading workflow, the variables to be used in Equation 6 are as follows:

- $|confirmed\ positive \cap recalled\ by\ workflow|$, the number of cases with a 1 in column **cp** and a 1 in column **dr_decision**.
- $|analysed|$, the number of cases that are 0 or 1 in column **dr_decision**.

For the nDRT(A3)+XR(A2) workflow, the variables to be used in Equation 6 are

- $|confirmed\ positive \cap recalled\ by\ workflow|$, the number of cases with a 1 in column **cp** and a 1 in column **ndrt_a3_xr_a2_decision**.
- $|analysed|$, the number of cases with a 0 or 1 in column **ndrt_a3_xr_a2_decision**.

Both CDRs will be assessed overall (across all sites/vendors).

The absolute difference between the CDR of the DR workflow (CDR_{DR}) and that of the nDRT(A3)+XR(A2) workflow ($CDR_{nDRT(A3)+XR(A2)}$) will be

$$Absolute\ difference = CDR_{nDRT(A3)+XR(A2)} - CDR_{DR}$$

It will be reported with its 90% Wilson score interval.

The relative difference between the two workflows will be

$$Relative\ difference = \frac{CDR_{nDRT(A3)+XR(A2)}}{CDR_{DR}}$$

and will be reported with its 90% confidence interval, as determined through bootstrapping (see Section 8.1.3 for details).

8.3. Secondary Endpoints

The secondary endpoints are denoted in Tables 2 and 3 with the letter S. For all metrics, the point estimate and 90% and 95% confidence intervals (calculated as the Wilson score interval) will be reported.

Endpoints will be calculated from the data according to the table below, and using the formulas at the start of Section 8.

Table 6: Details of each calculation. Variable names containing [workflow] and/or [mia OP] are generalisations of variables such as xr_decision_mia_a1, where the [workflow] can be replaced with any relevant workflow (in this example, the XR workflow) and [mia OP] can be replaced with any relevant Mia operating point (in this example OP A1). A slash (/) indicates two different structures for potential variable names. Usually this is one that includes results from the Mia reader and one that does not.

Endpoint	Formula	Necessary variables and values from the raw data	
AR	(1)	arbitrated	arb_[workflow] / arb_[workflow]_mia_[mia OP] = 1
		analysed	arb_[workflow] /<br>arb_[workflow]_mia_[mia OP] = 0<br>OR<br>arb_[workflow] /<br>arb_[workflow]_mia_[mia OP] = 1
RR	(2)	recall	[workflow]_decision_mia_[OP] / dr_decision = 1
		analysed	[workflow]_decision_mia_[OP] = 0<br>OR<br>[workflow]_decision_mia_[OP] = 1<br>OR<br>dr_decision = 0<br>OR<br>dr_decision = 1
Positive discordance rate	(3)	positive discordant	sa_decision_mia_[OP] = 1<br>AND<br>dr_decision = 0
		analysed	sa_decision_mia_[OP] = 1<br>OR<br>sa_decision_mia_[OP] = 0
RRpd	(4)	positive discordant	sa_decision_mia_[OP] = 1<br>AND<br>dr_decision = 0
		recalled	[workflow]_decision_mia_[OP] = 1
NFR	(5)	negative flagged	negative_flagged_[OP] = 1<br>OR<br>sa_decision_mia_OP = 0 AND r1_decision = 0
		analysed	ndrt_decision_mia_[OP] = 0<br>OR<br>ndrt_decision_mia_[OP] = 1

Endpoint	Formula	Necessary variables and values from the raw data	
CDR	(6)	confirmed positive	cp = 1
		recall	[workflow]_decision_mia_[OP]/dr_decision = 1
		analysed	[workflow]_decision_mia_[OP]/dr_decision = 0 OR [workflow]_decision_mia_[OP]/dr_decision = 1
SEN	(7)	confirmed positive	cp = 1
		recall	[workflow]_decision_mia_[OP] / dr_decision = 1
SPECnp	(9)	non-positive	np = 1
		no recall	[workflow]_decision_mia_[OP] / dr_decision = 0
PPV	(10)	confirmed positive	cp = 1
		recall	[workflow]_decision_mia_[OP] / dr_decision = 1

8.3.1. Subgroup analysis

Result for the endpoints involving the DR, nDRT, nIR, XR, nDRT+XR, nIR+XR workflows (Tables 2 and 3), the analyses will be repeated for the following subgroups:

- Prevalent scans (**inc_prev** = prev)
- Incident scans (**inc_prev** = inc)
- Age group (**age_at_case** variable), with age group buckets of 10 years, e.g.:
  - 50 ≤ **age_at_case** < 60,
  - 60 ≤ **age_at_case** < 70,
  - **age_at_case** ≥ 70.
- Breast density

8.3.2. Sensitivity of Overlapping Sets

For each of the following sets, the sensitivity will be calculated, per Formula 7 in Section 8:

Table 7: Details of the sensitivity calculation for each overlapping set.

Group	Necessary variables and values from the raw data	
Cancers detected by both R1 and Mia	confirmed positive	cp=1
	recall	sa_decision_mia_[OP] AND r1_decision = 1
Cancers detected R1 only, not Mia	confirmed positive	cp = 1
	recall	sa_decision_mia_[OP] = 0<br>AND<br>r1_decision = 1
Cancers detected by Mia only, not R1	confirmed positive	cp=1
	recall	sa_decision_mia_[OP] = 1<br>AND<br>r1_decision = 0
Cancers detected by both double reading with Mia and without Mia	confirmed positive	cp=1
	recall	[workflow]_decision_mia_[OP] AND dr_decision = 1
Cancers detected by double reading with Mia only	confirmed positive	cp = 1
	recall	[workflow]_decision_mia_[OP] = 1<br>AND<br>dr_decision = 0
Cancers detected by double reading without Mia only	confirmed positive	cp=1

Group	Necessary variables and values from the raw data	
	recall	[workflow]_decision_mia_[OP] = 0<br>AND<br>dr_decision = 1

8.3.3. Descriptives Of Cancer Subtypes Per Workflow And Among Agreed And Discordant Cancers Between Compared Workflows

For each workflow, DR, nDRT, nIR, XR, nDRT+XR, and nIR+XR, the number of cancers detected by the given workflow will be tabulated, along with their percentages (out of all cancers in the dataset), according to:

- Invasiveness
- Receptor status
- For invasive cancers:
  - Tumour size
  - Lymph node status
  - Histological grade
  - Tumour type

Additionally, information on cancer tumour size will be reported according to the summary statistics for continuous variables and/or based on the clinical used dichotomous classification of each variable (see Section 6.1).

8.3.4. WORKLOAD SAVINGS COMPARED TO DOUBLE READING WITHOUT MIA

For each workflow, nDRT, nIR, XR, nDRT+XR, and nIR+XR, the reduction in the number and proportion of cases required to be read by R2 will be calculated and reported.

This is calculated according to Table 8 below:

Table 8: Summary of how to determine which cases were read by Reader 2 for a given workflow. The OP is always the OP for the nDRT or nIR workflow.

Workflow	Necessary variables and values from the raw data	
nDRT	Not read by R2	negative_flagged_[OP] = 1<br>OR<br>sa_decision_mia_OP = 0 AND r1_decision = 0
	Read by R2	negative_flagged_[OP] = 0<br>OR<br>sa_decision_mia_OP = 1 OR r1_decision = 1
nIR	Not read by R2	sa_decision_mia_[OP] = r1_decision
	Read by R2	sa_decision_mia_[OP] ≠ r1_decision

The reduction in number of cases read by R2 compared to double reading is simply the number not read by R2. The reduction in the proportion of cases is simply the number not read by R2 divided by the total number of cases (the sum of those read by R2 and not read by R2).

The reduction of the number of arbitrations required to be done will be calculated by taking the number of cases arbitrated in the DR workflow (**arb_dr** = 1) and subtracting from it the number of arbitrations to be done in the other given workflow (**arb_[workflow]_mia_[OP] = 1**). The proportional reduction in arbitrations will be the arbitration rate of the DR workflow, AR_{DR}, divided by the arbitration rate of the comparator workflow, $AR_{workflow}$, minus 1. That is,

$$\frac{AR_{DR}}{AR_{workflow}} - 1.$$

This proportional reduction in the number of arbitrations required to be done will be used to calculate overall workload savings if arbitration read time takes 1x, 2x, 3x, or 4x first or second reads. The time savings will be the proportion multiplied by 1/3, 1/2, 3/5, and 2/3, respectively.

Measured arbitration read times will also be used to calculate overall workload savings. This will be performed by multiplying the time savings by the mean read time for a given workflow and operating point and by using the actual read time measurements as available.

8.3.5. Descriptives Of Cancer Subtypes Per Single Reader And Among Agreed And Discordant Cancers Between Single Readers

For the single readers, Mia, R1 and R2, the number of cancers detected by each will be tabulated, along with their percentages (out of all cancers in the dataset), according to:

- Invasiveness
- Receptor status
- For invasive cancers:
    - Tumour size
    - Lymph node status
    - Histological grade
    - Tumour type

Additionally, information on cancer tumour size will be reported according to the summary statistics for continuous variables (see Section 5.1).

There will also be a table reporting on all of the endpoints above, but restricted to cases where both readers agreed to recall/not recall. There will be another table reporting on these endpoints in cases where the readers did not agree.

8.4. Exploratory Endpoints

For the endpoints related to single readers R1, R2, and Mia Standalone (Table 2), the analyses will be repeated for the following subgroups:

- Prevalent scans (**inc_prev** = prev)
- Incident scans (**inc_prev** = inc)
- Age group (**age_at_case** variable), with age group buckets of 10 years, e.g.:
  - 50 ≤ **age_at_case** < 60,
  - 60 ≤ **age_at_case** < 70,
  - **age_at_case** ≥ 70.
- Breast density

Section 9. List of Tables, Listings and Figures

Per agreement with the sponsor, the following endpoints will be presented as tables.

Tables	
14.1.1	Demographics
14.1.2	Evidence class counts per age group
14.2.1	Absolute and relative difference of CDR of the double reader and the nDRT(A3)+XR(A2) workflows, assessed for non-inferiority and superiority
14.2.2	Arbitration Rates per workflow
14.2.3	Negative Flag Rates for Mia nDRT workflow
14.2.4	Positive and negative discordance rates per workflow and OPs
14.2.5	Recall rates per workflow
14.2.6	Recall rates among positive discordant cases per workflow and OPs
14.2.7	Cancer detection rates per workflow
14.2.8	Sensitivity per workflow
14.2.9	Sensitivity of overlapping sets
14.2.10	Positive predictive value per workflow
14.2.11	Specificity on non-positives per workflow
14.2.12	Reduction in portion of cases read by R2 (compared to DR)
14.2.13	Read times (in seconds)
14.2.14	Workload Savings Compared to Double Reading Without Mia
14.2.15	Reduction in portion of cases read by an arbitrator (compared to DR)
14.2.16	Workload savings based on measured reading times and if Arb read time is 1x, 2x, 3x, 4x first reads time
14.2.17	Descriptives of cancer subtypes, per workflow
14.2.18	Descriptives of cancer subtypes in invasive cancers, per workflow
14.2.19	Descriptives of cancer subtypes among positive discordant cases, per workflow
14.2.20	Subgroup analysis Mia SA metrics
14.2.21	Subgroup analysis R1, R2, and DR metrics
14.2.22	Subgroup analysis Mia nDRT metrics
14.2.23	Subgroup analysis Mia XR metrics

14.2.24	Subgroup analysis Mia nDRT+XR metrics
14.2.25	Subgroup analysis Mia nIR+XR metrics
14.2.26	Non-inferiority and superiority test results for Mia nDRT+XR workflow at OP A2+A1 against DR
14.2.27	Non-inferiority and superiority test results for Mia nDRT+XR workflow at OP A2+A2 against DR
14.2.28	Non-inferiority and superiority test results for Mia nDRT+XR workflow at OP A3+A1 against DR
14.2.29	Non-inferiority and superiority test results for Mia nDRT+XR workflow at OP A3+A2 against DR
14.2.30	Non-inferiority and superiority test results for Mia nDRT+XR workflow at OP A4+A1 against DR
14.2.31	Non-inferiority and superiority test results for Mia nDRT+XR workflow at OP A4+A2 against DR
14.2.32	Non-inferiority and superiority test results for Mia nIR+XR workflow at OP A1+A1 against DR
14.2.33	Non-inferiority and superiority test results for Mia nIR+XR workflow at OP A1+A2 against DR
14.2.34	Non-inferiority and superiority test results for Mia nIR+XR workflow at OP A2+A1 against DR
14.2.35	Non-inferiority and superiority test results for Mia nIR+XR workflow at OP A2+A2 against DR

REFERENCES

[1] Kheiron Medical Technologies; Grampian Health Board & University of Aberdeen, "GEMINI: Grampian's Evaluation of Mia an Innovative National breast screening Initiative," Kheiron Medical Technologies Ltd., 2023.

[2] CPMP/ICH/363/96, "Statistical Principles for Clinical Trials, ICH Guideline Topic E9; Note for Guidance on Statistical Principles for Clinical Trials," September 1998.

[3] Quantics, "SOP S1 DSCP and single programming".

[4] Quantics, "SOP S3 Planning a statistical analysis".

[5] R Core Team, "R: A Language and Environment for Statistical Computing," Vienna, Austria, 2015.

[6] G. van Rossum and F. Drake, "Python 3 Reference Manual," CreateSpace, Scotts Valley, CA, 2009.

[7] L. Brown, T. Cai and A. DasGupta, "Interval Estimation for a Binomial Proportion," *Statistical Science,* vol. 16, no. 2, pp. 101-133, 2001.

[8] R. Newcombe, "Interval estimation for the difference between independent proportions: comparison of eleven methods," *Statistics in Medicine,* vol. 17, no. 8, pp. 873-890, 1998.

2847_Kheiron_KMT008_SAP_v_2_0 Clean (Aberdeen) (1)

Final Audit Report 2024-01-11

Created:	2023-12-22
By:	Adam Heroux (adam@kheironmed.com)
Status:	Signed
Transaction ID:	CBJCHBCAABAAnpglwDTvdbE-SXbGl4BpUKCygFulJLpA

"2847_Kheiron_KMT008_SAP_v_2_0 Clean (Aberdeen) (1)" History

Document created by Adam Heroux (adam@kheironmed.com)
2023-12-22 - 7:19:58 PM GMT

Document emailed to lesley.anderson@abdn.ac.uk for signature
2023-12-22 - 7:25:17 PM GMT

Document emailed to annie@kheironmed.com for signature
2023-12-22 - 7:25:17 PM GMT

Document emailed to clarisse.devries@abdn.ac.uk for signature
2023-12-22 - 7:25:17 PM GMT

Document emailed to gerald.lip@nhs.scot for signature
2023-12-22 - 7:25:18 PM GMT

Email viewed by annie@kheironmed.com
2023-12-22 - 7:27:00 PM GMT

Signer annie@kheironmed.com entered name at signing as Annie Ng
2023-12-22 - 7:27:52 PM GMT

Document e-signed by Annie Ng (annie@kheironmed.com)
Signature Date: 2023-12-22 - 7:27:54 PM GMT - Time Source: server

Email viewed by lesley.anderson@abdn.ac.uk
2024-01-08 - 8:38:26 AM GMT

Email viewed by gerald.lip@nhs.scot
2024-01-08 - 11:11:33 AM GMT

New document URL requested by gerald.lip@nhs.scot

2024-01-08 - 11:11:38 AM GMT

Email viewed by clarisse.devries@abdn.ac.uk

2024-01-08 - 11:18:20 AM GMT

New document URL requested by clarisse.devries@abdn.ac.uk

2024-01-08 - 11:21:47 AM GMT

Signer clarisse.devries@abdn.ac.uk entered name at signing as Clarisse de Vries

2024-01-08 - 11:23:13 AM GMT

Document e-signed by Clarisse de Vries (clarisse.devries@abdn.ac.uk)

Signature Date: 2024-01-08 - 11:23:15 AM GMT - Time Source: server

Email viewed by lesley.anderson@abdn.ac.uk

2024-01-09 - 12:01:08 PM GMT

Signer lesley.anderson@abdn.ac.uk entered name at signing as Professor Lesley Anderson

2024-01-09 - 12:01:47 PM GMT

Document e-signed by Professor Lesley Anderson (lesley.anderson@abdn.ac.uk)

Signature Date: 2024-01-09 - 12:01:49 PM GMT - Time Source: server

Signer gerald.lip@nhs.scot entered name at signing as Gerald Lip

2024-01-11 - 9:39:03 AM GMT

Document e-signed by Gerald Lip (gerald.lip@nhs.scot)

Signature Date: 2024-01-11 - 9:39:05 AM GMT - Time Source: server

Agreement completed.

2024-01-11 - 9:39:05 AM GMT
