# Supplementary material for: Prospective evaluation of artificial intelligence integration into breast cancer screening in multiple workflow settings: the GEMINI study
Source: Nat Cancer. 2026 Mar 10;7(3):484–93. doi: 10.1038/s43018-026-01126-1 (PMC13035467; doi:10.1038/s43018-026-01126-1)
Supplement: Supplementary file 2 — Reporting Summary [file 43018_2026_1126_MOESM2_ESM.pdf]

nature portfolio

Corresponding author(s): Clarisse F. de Vries
Last updated by author(s): Nov 4, 2025

Reporting Summary

Nature Portfolio wishes to improve the reproducibility of the work that we publish. This form provides structure for consistency and transparency in reporting. For further information on Nature Portfolio policies, see our Editorial Policies and the Editorial Policy Checklist.

Statistics

For all statistical analyses, confirm that the following items are present in the figure legend, table legend, main text, or Methods section.

n/a	Confirmed	
☐	☒	The exact sample size (*n*) for each experimental group/condition, given as a discrete number and unit of measurement
☒	☐	A statement on whether measurements were taken from distinct samples or whether the same sample was measured repeatedly
☐	☒	The statistical test(s) used AND whether they are one- or two-sided<br>*Only common tests should be described solely by name; describe more complex techniques in the Methods section.*
☐	☒	A description of all covariates tested
☐	☒	A description of any assumptions or corrections, such as tests of normality and adjustment for multiple comparisons
☐	☒	A full description of the statistical parameters including central tendency (e.g. means) or other basic estimates (e.g. regression coefficient) AND variation (e.g. standard deviation) or associated estimates of uncertainty (e.g. confidence intervals)
☐	☒	For null hypothesis testing, the test statistic (e.g. *F*, *t*, *r*) with confidence intervals, effect sizes, degrees of freedom and *P* value noted<br>*Give P values as exact values whenever suitable.*
☒	☐	For Bayesian analysis, information on the choice of priors and Markov chain Monte Carlo settings
☒	☐	For hierarchical and complex designs, identification of the appropriate level for tests and full reporting of outcomes
☒	☐	Estimates of effect sizes (e.g. Cohen's *d*, Pearson's *r*), indicating how they were calculated

Our web collection on statistics for biologists contains articles on many of the points above.

Software and code

Policy information about availability of computer code

Data collection	A commercially available AI system (MiaTM v3; Kheiron Medical Technologies Ltd) was prospectively utilised within a routine breast screening service. Based on a woman's screening mammograms, it outputs a continuous malignancy prediction value ranging from 0 to 1. The AI indicates that a woman should be invited back for additional examinations if her mammograms' malignancy prediction value is above a certain decision threshold.
Data analysis	Statistical analyses were performed in R (version 4.2.1). The statistical output alongside the relevant R code is available in the Appendix. In addition, the R code used for statistical analysis can be found in the following GitHub repository: https://github.com/CFdeVries/gemini-paper. DOI: https://doi.org/10.5281/zenodo.14826292.

For manuscripts utilizing custom algorithms or software that are central to the research but not yet described in published literature, software must be made available to editors and reviewers. We strongly encourage code deposition in a community repository (e.g. GitHub). See the Nature Portfolio guidelines for submitting code & software for further information.

Data

Policy information about availability of data

All manuscripts must include a data availability statement. This statement should provide the following information, where applicable:

- Accession codes, unique identifiers, or web links for publicly available datasets
- A description of any restrictions on data availability
- For clinical datasets or third party data, please ensure that the statement adheres to our policy

SBSS images and data are not publicly available. Access to the raw, de-identified SBSS data and mammograms is subject to the required approvals by the Data Protection Officer (DPO), Caldicott Guardian, and other required data agreements being in place. Data access requests are typically reviewed and approved within approximately 6-8 weeks. More information can be found on the DaSH website: https://www.abdn.ac.uk/research/digital/platforms/safe-haven-dash/." The Evaluation Plan and the Statistical Analysis Plan are available in the Supplementary Information. Source data for Figure 3 has been provided as a Source Data file.

Research involving human participants, their data, or biological material

Policy information about studies with human participants or human data. See also policy information about sex, gender (identity/presentation), and sexual orientation and race, ethnicity and racism.

Reporting on sex and gender	The source population for the GEMINI study included women attending routine breast screening within NHS Grampian, part of the Scottish Breast Screening Service (SBSS), who did not opt-out of the study and whose mammograms were read between 27 Feb 2023 and 05 October 2023. SBSS uses the Community Health Index (CHI) number, a unique patient identifier used in Scotland, to identify women. The AI read mammograms from screening attendees identified as female in the DICOM data.
Reporting on race, ethnicity, or other socially relevant groupings	Race/ethnicity is not routinely recorded in the NHS Grampian breast screening centre, so could not be reported in the paper.
Population characteristics	The UK National Health Service Breast Screening Programme (NHS-BSP) invites women aged 50 to 71 years, every 3 years, for routine breast screening using digital mammography. Further demographic information is provided in Table 1.
Recruitment	The source population for the GEMINI study included women attending routine breast screening within NHS Grampian, part of the Scottish Breast Screening Service (SBSS), who did not opt-out of the study and whose mammograms were read between 27 Feb 2023 and 05 October 2023. Opt-out rather than opt-in limited the potential for self-selection bias, as did the low opt-out rate of 0.5%.
Ethics oversight	This study was considered a service evaluation [NHS Grampian Quality Improvement and Assurance (ID number: 5834)] by the local Research Governance team and did not require ethical approval. The evaluation was registered with the Scottish National Screening Organisation Research and Innovation group and received NHS Grampian Caldicott Approval.

Note that full information on the approval of the study protocol must also be provided in the manuscript.

Field-specific reporting

Please select the one below that is the best fit for your research. If you are not sure, read the appropriate sections before making your selection.

☒ Life sciences ☐ Behavioural & social sciences ☐ Ecological, evolutionary & environmental sciences

For a reference copy of the document with all sections, see nature.com/documents/nr-reporting-summary-flat.pdf

Life sciences study design

All studies must disclose on these points even when the disclosure is negative.

Sample size	The sample size was based on a non-inferiority test for the relative difference between the routine double reading workflow and the primary AI workflow in detecting screen-detected cancers. The agreement rate between the routine double reading workflow and the primary AI workflow was expected to be 95.0%. A percentage of 1.9% of the confirmed positives was expected to only be detected by the routine double reading workflow, while 3.0% was expected to be detected by the AI workflow. Using a one-sided alpha of 0.05 and a non-inferiority margin of 10% relative to the routine double reading workflow proportion, a sample size of 65 confirmed positives was determined before study commencement to have a power of 91.5%. Achieving this sample size ensures that the power for the secondary endpoints for the AI workflow is at least 90%. Due to natural variation in cancer detection rate and the time duration between mammography assessment and potential cancer diagnosis, the study conclusion date was estimated to allow 65 confirmed positives to be achieved, resulting in 106 confirmed positives in this sample.
Data exclusions	The UK National Health Service Breast Screening Programme (NHS-BSP) invites women aged 50 to 71 years, every 3 years, for routine breast screening using digital mammography. Between 27 February and 5 October 2023, 17,421 women attended routine screening at the NHS Grampian (Aberdeen, Scotland). Of these, 93 (0.5%) women opted out of the study, and 175 (1.0%) technical recalls were excluded. Images from 4,992 (29.1%) women were not sent for AI assessment due to an unanticipated legacy IT coding error, initiating a mammographer session timeout after 30 minutes of inactivity. The AI system did not read 1,260 (10.4%) mammogram examinations due to its

	exclusion criteria.<br><br>Full details of exclusions can be found in Figure 1.
Replication	The DaSH team pseudonymised and provisioned the dataset into a secure DaSH workspace, accessible to the NHS Grampian & University of Aberdeen study team only (CFDV, JAD, GL & LAA). The AI vendor could not access this workspace to ensure the evaluation was performed independently from the industry partner.<br><br>The AI vendor was given access to the data within a separate workspace, where they established the accuracy of the reported results.
Randomization	There was no randomization as part of this study.
Blinding	There was no group allocation during the study, so blinding was not relevant. The triaging functionality of the AI, which would separate women into groups, was simulated.<br><br>The AI results (recall opinion and regions of interest) were not available to the human readers during the standard reading and arbitration process. These results were only released to the readers when the AI suggested to recall but routine screening did not.

Reporting for specific materials, systems and methods

We require information from authors about some types of materials, experimental systems and methods used in many studies. Here, indicate whether each material, system or method listed is relevant to your study. If you are not sure if a list item applies to your research, read the appropriate section before selecting a response.

Materials & experimental systems

n/a	Involved in the study
☒	☐ Antibodies
☒	☐ Eukaryotic cell lines
☒	☐ Palaeontology and archaeology
☒	☐ Animals and other organisms
☒	☐ Clinical data
☒	☐ Dual use research of concern
☒	☐ Plants

Methods

n/a	Involved in the study
☒	☐ ChIP-seq
☒	☐ Flow cytometry
☒	☐ MRI-based neuroimaging

Plants

Seed stocks	*Report on the source of all seed stocks or other plant material used. If applicable, state the seed stock centre and catalogue number. If plant specimens were collected from the field, describe the collection location, date and sampling procedures.*
Novel plant genotypes	*Describe the methods by which all novel plant genotypes were produced. This includes those generated by transgenic approaches, gene editing, chemical/radiation-based mutagenesis and hybridization. For transgenic lines, describe the transformation method, the number of independent lines analyzed and the generation upon which experiments were performed. For gene-edited lines, describe the editor used, the endogenous sequence targeted for editing, the targeting guide RNA sequence (if applicable) and how the editor was applied.*
Authentication	*Describe any authentication procedures for each seed stock used or novel genotype generated. Describe any experiments used to assess the effect of a mutation and, where applicable, how potential secondary effects (e.g. second site T-DNA insertions, mosiacism, off-target gene editing) were examined.*
